# Transcription Factors AhR/ARNT Regulate the Expression of *CYP6CY3* and *CYP6CY4* Switch Conferring Nicotine Adaptation

**DOI:** 10.3390/ijms20184521

**Published:** 2019-09-12

**Authors:** Yiou Pan, Tianfei Peng, Pengjun Xu, Xiaochun Zeng, Fayi Tian, Jiabao Song, Qingli Shang

**Affiliations:** 1 School of Agricultural Science, Zhengzhou University, Zhengzhou 450001, China; 2 College of Plant Science, Jilin University, Changchun 130062, China; 3 Institute of Tobacco Research, Chinese Academy of Agricultural Sciences, Qingdao 266101, China

**Keywords:** cytochrome P450, gene regulation, element, host adaptation

## Abstract

Nicotine is one of the most toxic secondary plant metabolites in nature and it is highly toxic to herbivorous insects. The overexpression of *CYP6CY3* and its homologous isozyme *CYP6CY4* in *Myzus persicae nicotianae* is correlated with nicotine tolerance. The expanded (AC)_n_ repeat in promoter is the *cis* element for *CYP6CY3* transcription. These repeat sequences are conserved in the *CYP6CY3* gene from *Aphis gossypii* and the homologous P450 genes in *Acyrthosiphon pisum*. The potential transcriptional factors that may regulate *CYP6CY3* were isolated by DNA pulldown and sequenced in order to investigate the underlying transcriptional regulation mechanism of *CYP6CY3*. These identified transcriptional factors, *AhR* and *ARNT*, whose abundance was highly correlated with an abundance of the *CYP6CY3* gene, were validated. RNAi and co-transfection results further confirm that *AhR* and *ARNT* play a major role in the transcriptional regulation of the *CYP6CY3* gene. When the *CYP6CY3* transcript is destabilized by *AhR/ARNT* RNAi, the transcription of the *CYP6CY4* is dramatically up-regulated, indicating a compensatory mechanism between the *CYP6CY3* and *CYP6CY4* genes. Our present study sheds light on the *CYP6CY3* and *CYP6CY4* mediated nicotine adaption of *M. persicae nicotianae* to tobacco. The current studies shed light on the molecular mechanisms that underlie the genotypic and phenotypic changes that are involved in insect host shifts and we conclude that AhR/ARNT regulate the expression of *CYP6CY3* and *CYP6CY4* cooperatively, conferring the nicotine adaption of *M. persicae nicotianae* to tobacco

## 1. Introduction

*Myzus persicae* Sulzer (Hemiptera: Aphididae) is one of the most destructive crop pests globally. It causes damage to various host plants directly via direct feeding, plant virus transmission through feeding, and indirectly through honeydew contamination [1]. The ability of *M. persicae* to adapt to new host plants has led to the formation of host races. The best-documented race is associated with tobacco and it is called *M. persicae nicotianae* [2]. Although morphologically and genetically different from *M. persicae sensu stricto* (s.s.), there is clear evidence of gene flow between the two taxa [3]. Research has shown that *M. persicae* clones that were collected from Western Australia are successfully adapted to *Lupinus angustifolius*, and the improved tolerance of lupanine in their diet might confer this adaptation as compared to non-adapted clones [4].

Insects have employed cytochrome P450 monooxygenases (P450s) that promote more a rapid metabolism to defer the cost of a counter-defence against toxic plant compounds [5,6,7,8,9,10,11]. Members of the CYP3 clade have been implicated in the oxidative detoxification of furanocoumarins, alkaloids, and numerous other secondary plant metabolites and synthetic insecticides [12,13,14,15]. In tobacco-adapted *M. persicae*, *CYP6CY3* overexpression plays a detoxifying role, protecting the insect from the plant’s secondary metabolite nicotine [16,17]. Additionally, the constitutive overexpression of *CYP6CY3* in *M. persicae nicotianae* as compared to *M. persicae s.s*, which is partly due to gene amplification, accounts for neonicotinoid resistance [16,18].

Currently, regulatory cascades in insects still largely remain a mystery. One allelochemical regulatory cascade, the xanthotoxin cascade in insects, has been well studied. In this cascade, an overlapping ecdysone response element (EcRE), antioxidant response element (ARE), xenobiotic response element to xanthotoxin (XRE-xan), and a xenobiotic response element to an aryl hydrocarbon receptor (XRE-AhR) have been functionally characterized from both the *CYP6B1* and *CYP6B4* promoters of *Papilio polyxenes* and *P. glaucus*, respectively [19,20,21,22]. The *AhR* ortholog, spineless (*Ss*), and the aryl hydrocarbon receptor nuclear translocator (*ARNT*) orthologue, tango (*Tgo*), both bind to XRE-AhR, which enhances the basal expression of *CYP6B1* but not the magnitude of xanthotoxin and benzopyrene induction [19].

To date, there have been no reports on how transcriptional factors regulate *CYP6CY3*. We chose to study the regulatory cascades of *CYP6CY3*, which is related to nicotine tolerance [16,18]. In this study, we identified an element in the *CYP6CY3* promoter and the transcription factors that transcriptionally regulate *CYP6CY3*. We also identified that the closely related P450 becomes more activated under certain circumstances. The present study sheds light on the *CYP6CY3* and *CYP6CY4* mediated nicotine adaption of *M. persicae nicotianae* to tobacco. In *Aphis gossypii*, the homologous conserved *CYP6CY3* was regulated by a similar mechanism.

## 2. Results

### 2.1. Knockdown of CYP6CY3 and CYP6CY4 Together Significantly Increases Nicotine Toxicity in M. Persicae Nicotianae

**Figure 1:**
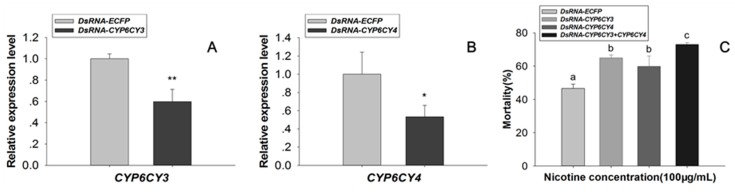
dsRNA-mediated suppression of *CYP6CY3* and *CYP6CY4* transcripts and its effect on nicotine toxicity in *M. persicae nicotianae*. (**A**,**B**) dsRNA oral-mediated knockdown efficacy (100 ng/μL dsRNA-*CYP6CY3* or dsRNA-*CYP6CY4*) after 48 h and the relative *CYP6CY3* and *CYP6CY4* expression levels in *M. persicae nicotianae* orally treated with dsRNA [23]. The housekeeping genes *actin* and *para* were used as internal reference genes [24]. (**C**) Mean mortality ± SE (*n* = 3) after feeding on a nicotine (100 mg/L) and dsRNA mixture (100 ng/μL of dsRNA-*CYP6CY3* or dsRNA-*CYP6CY4* or dsRNA-*CYP6CY3*+*CYP6CY4*) for 48 h in *M. persicae nicotianae*. Error bars indicate 95% confidence intervals (*n* = 3). Different letters on the bars of the histogram indicate significant differences based on ANOVA, followed by Tukey’s HSD multiple comparison test (*p* < 0.05). * Significant difference by Student’s *t*-test (*p* < 0.05). ** Significant difference by Student’s *t*-test (*p* < 0.01).

The expressions of *CYP6CY3* and the homologous isozyme *CYP6CY4* detected in previous transcriptome data (Supplementary Data S1, the transcriptome data were deposited in the National Center for Biotechnology Information/Sequence Read Archive (NCBI/SRA) database, with a SRA accession number SRX1499035 [23]) were consistent with the qPCR results reported by Peng et al. (2016a) [23]. Knocking down the expression of *CYP6CY3* and *CYP6CY4* together significantly increased the mortality *M. persicae nicotianae* (Green) (its mortality increased 30.30% in dsRNA-*CYP6CY3*+*CYP6CY4*-fed aphids) under nicotine stress, as compared with the control (Figure 1). Silencing both *CYP6CY3* and *CYP6CY4* together was more effective than just silence one P450 gene [23].

### 2.2. Characterization of the Element in the CYP6CY3 Promoter Sequence

**Figure 2:**
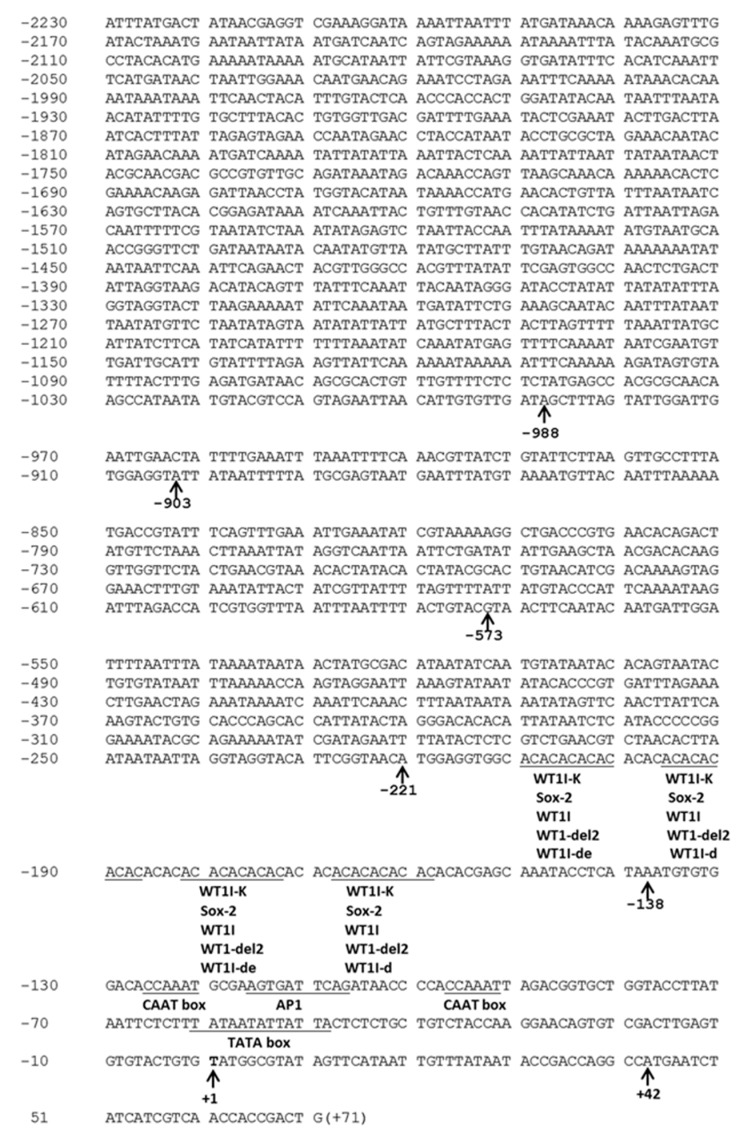
The promoter, 5′-UTR and 5′-most coding sequences of *CYP6CY3* in *M. persicae*. The nucleotides are numbered relative to the transcription start site (TSS), indicated by +1, with upstream sequences preceded by “-” and downstream sequences by “+”. The TATA box and other putative *cis* elements are underlined. The start codon ATG is shown in bold. All of the left and right boundaries of the constructs made in this study are marked by an arrow and are numbered below the corresponding nucleotides.

The different lengths of the resulting PCR bands that were obtained from the *M. persicae nicotianae* genomic DNA digested with AfeI, EcoRV-HF, PvuII, and PmeI while using genome walking were sequenced. The sequencing results showed that all of the PCR fragments were 5′-flanking *CYP6CY3* promoter sequences with different lengths in the 5′-flanking region (Genebank No. KF998221, KF998222, KF998223, KF998224, and KF998225). The longest 5′-flanking promoter sequence was derived from AfeI-digested genomic DNA (Figure 2, Genebank No. KF998221).

All of these sequences encoded the first 29 bp of the *CYP6CY3* coding sequence and 42 bp of the 5′ UTR, but with different (AC)_n_ repeat lengths. The nucleotides are numbered relative to the transcription start site (TSS) at +1. The upstream sequences are preceded by “-”, and the downstream sequences are indicated by “+”. The sequence analysis indicated typical characteristics, such as the TATA box located at -48~-61 bp and two CAAT boxes located at the -92~-97 bp and -120~-126 bp regions. A core upstream promoter element that was located at -138~-221 bp, which was rich in (AC)_n_ repeats, was predicted by constructing matrices on the fly from TRANSFAC 4.0 sites using TRANSFAC 4.0 software in the AliBaba 2.1 database (Pairsim to know sites value is 64, the match width in bp value is 10, the minimum number of sites is 5, the minimum match conservation value is 75%, the similarity of between the sequence and match value is 100%, and the factor class level is 4). Although the putative element may or may not be a functional element in the *CYP6CY3* promoter, we considered its position when we made progressive and internal deletion/mutation constructs. The homology BLAST results indicated that the *CYP6CY3* sequence (Genbank No. HM009309) shared 88.19% of its identity with the *CYP6CY3* from *Acyrthosiphon pisum* at the amino acid level (NCBI Reference Sequence: NM_001366197.1). Further analysis illustrated that the *CYP6CY3* promoter sequence in *A. pisum* also contained an (AC)_n_ repeat region, but it showed great variation in other regions (Figure S1). Two gene specific primers amplified the *CYP6CY3* promoter of *A. gossypii* (New GSP1 and New GSP2) by using genome walking, and the sequencing results indicated that *A. gossypii* has a promoter with the same characterization in its *CYP6CY3* (Figure S2). The *CYP6CY*4 promoter from *M. persicae* that was obtained from the genome walker did not exhibit these characteristics (Figure S3).

**Figure 3:**
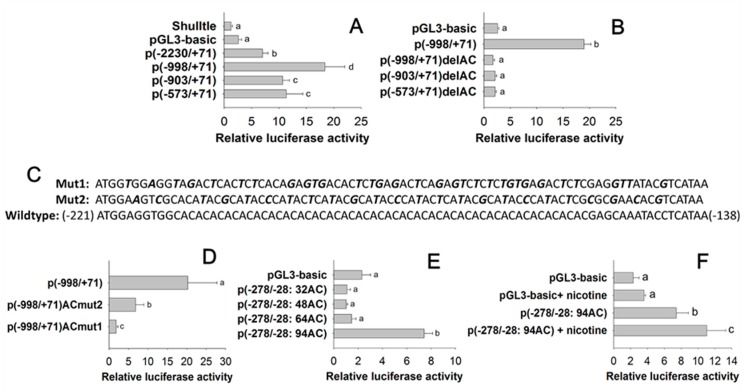
Mapping of core *cis* elements in the *CYP6CY3* promoter region by 5′ progressive deletions (**A**), internal deletions (**B**) and mutations (**C**,**D**), and the effect of (AC)_n_ repeat length on promoter activity (**E**) and its nicotine-inducible activity (**F**).The name of each *CYP6CY3* promoter-pGL3 construct contains a “P” and a pair of parentheses with two numerals separated by a dash to specify the 5′ and 3′ positions of the corresponding promoter fragment. For internal deletion or substitution mutation constructs, the name of the deleted or mutated sequence and a “-del” or “-mut” are added after the parentheses. The deleted region was ~-221–-138 as shown in Figure S2. The mutated nucleotides in the (AC)_n_ repeats are bold and shown in italics. The final concentration of nicotine for induction was 24.7 μM/L. Different letters on the bars of the histogram indicate significant differences based on an ANOVA, followed by Tukey’s HSD multiple comparison test (*p* < 0.05).

A set of eight peach aphid *CYP6CY3* promoter 5′ progressive deletion constructs [p(-2230/+71), p(-998/+71), p(-903/+71), and p(-573/+71)] (see the boundaries in Figure 2) were co-transfected into Sf9 cells with a phRL-TK control plasmid to roughly define the regions harboring a core *cis* element. Relative to the p(-2230/+71) construct, the progressive 5′ deletions to -998 significantly increased the basal promoter activity, whereas the 5′ deletion to -903 and -578 marginally increased the promoter activity (Figure 3A). When compared with p(-998/+71), p(-903/+71), and p(-573/+71), the internal deletion of AC repeats [p(-998/+71)delAC, p(-903/+71)delAC, and p(-573/+71)delAC] completely depressed the basal promoter activity (Figure 3B). Further mutation indicated that the nearly complete removal of the (AC)_n_ repeat region [p(-998/+71)ACmut2] abolished promoter activity, whereas the incomplete (AC)_n_ repeat mutation [p(-998/+71)ACmut1] significantly decreased promoter activity when compared with the p(-998/+71) construct (Figure 3C,D).

Sequencing the DNA fragments from promoters (-278/-28) that contain AC repeat regions revealed at least five *CYP6CY3* copies (with different AC repeat length: 94-, 84-, 64-, 48-, and 32-bp AC repeats) in the genome of *M. persicae nicotianae*. The transfection results indicate that the (AC)_n_ repeat expansion influenced the transcriptional activity of its promoter until it reached up to 94 bp, and the other (AC)_n_ repeats (64-, 48-, and 32-bp AC repeats) only confer lower basal transcription activity for their promoters (Figure 3E). The activity of p(-278/-28: 94AC) was inducible by nicotine, as compared to the control (Figure 3F).

### 2.3. Identification of Core Cis Element Binding Proteins

The proteins isolated by DNA pulldown were analysed via capillary high-performance liquid chromatography-mass spectral analysis (CE/HPLC-MS; Aptbiotech Co., Ltd., Shanghai, China) to identify the *cis* element that binds the nuclear proteins. A total of 955 proteins were positively identified (Supplementary Data S2). These identified proteins included many non-target proteins and transcription related proteins, such as DNA-directed RNA polymerase, RNA recognition protein, pre-mRNA processing factor, putative RNA polymerase II elongator, and DNA topoisomerase. The transcription factor proteins were also isolated, including the aryl hydrocarbon receptor (AhR), the putative c2h2-type zn-finger protein (CncC), and the cyclic AMP-dependent transcription factor (Camp).

**Figure 4:**
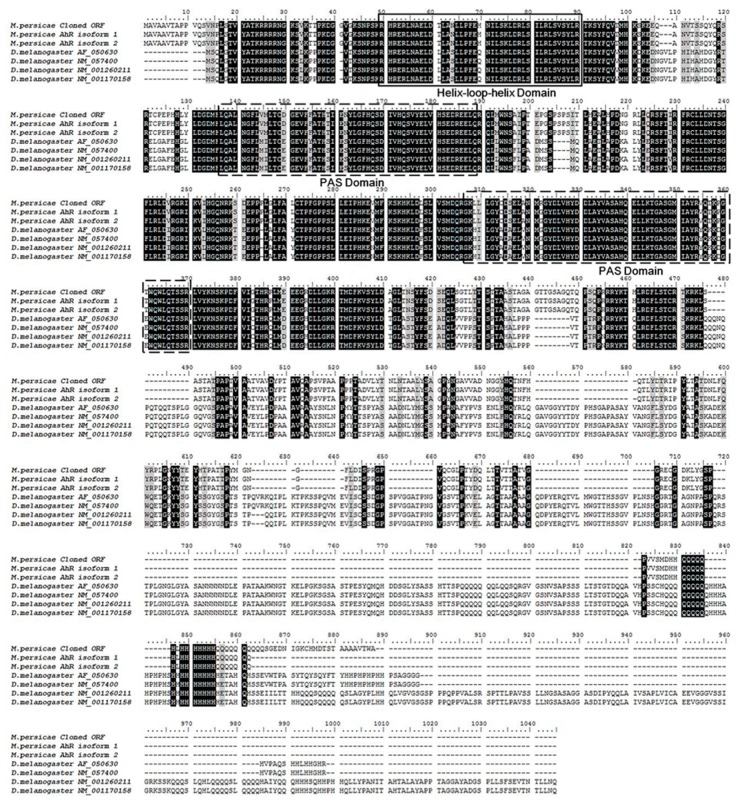
Alignment of the amino acid sequences of *AhR* of insect species. The amino acid sequence of *AhR* of *M*. *persicae* shares a high level of homology with that of *D. melanogaster*. The conserved domains common to AhRs are boxed, including the basic helix-loop-helix (bHLH) and PER-ARNT-SIM (PAS) specific to *AhR* members. The bHLH region is involved in the binding of the transcription factor to DNA and facilitates protein-protein interactions. The PAS domains support specific secondary interactions with other PAS domain containing proteins, as is the case with AhR and ARNT. The glutamine-rich (Q-rich) domain is located in the C-terminal region of the protein and it is involved in co-activator recruitment and transactivation The *AhR* sequences of *D. melanogaster* were downloaded from Gene Bank with the accession number: AF050630, NM057400, NM001260211 and NM001170158. The *AhR* sequences of *M. persicae* were got from the transcriptome with the unigene number of c18023_g2_i1 and c18023_g2_i2 for *AhR* isofrom 1 and *AhR* isofrom 2, respectively (The clean reads obtained in this study were submitted to the NCBI/SRA database, SRA experiment accession number: SRX1499035).

**Figure 5:**
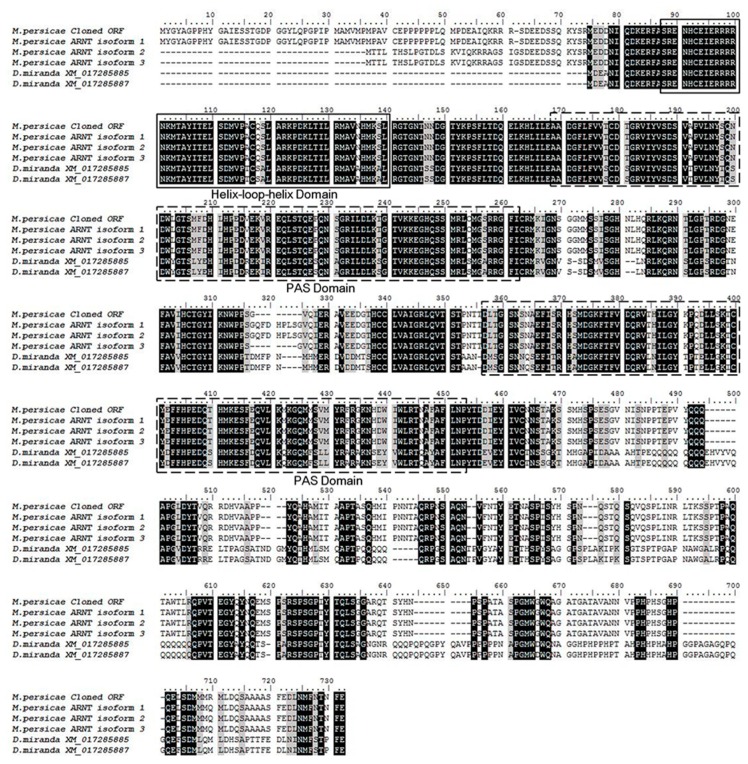
Alignment of the amino acid sequences of *ARNT* of insect species. The amino acid sequence of *ARNT* of *M*. *persicae* shares a high level of homology with that of *D. miranda*. The conserved basic helix-loop-helix (bHLH) and PER-ARNT-SIM (PAS) domains specific to *ARNT* members are boxed. The *ARNTs* Gene Bank number of *D. miranda* were XM017285885 and XM017285887. The corresponding unigene numbers of *ARNT* isofrom 1, *ARNT* isofrom 2 and *ARNT* isofrom 3 of *M. persicae* were c16053_g1_i1, c16053_g1_i2 and c16053_g1_i3, respectively (SRA experiment accession number: SRX1499035) [23].

The *AhR* and *ARNT* sequence were obtained from the transcriptome of the *M. persicae* (The National Center for Biotechnology Information/Sequence Read Archive (NCBI/SRA) database, SRA experiment accession number: SRX1499035) [23]. The alignment of the *AhR* and *ARNT* of *M. persicae* with the paralogs genes of *Drosophila* indicated that they share the same basic highly conserved domain, helix-loop-helix (bHLH), and the period (Per)-ARNT- single-minded (Sim) (PER-ARNT-SIM, PAS) (Figure 4 and Figure 5). The bHLH domain participates in the binding of the transcription factors to the DNA and it promotes protein-protein interaction, and the PAS domain supports specific secondary interactions with other PAS domains of AHR and ARNT [25,26]. The glutamine-rich (Q-rich) domain is located in the C-terminal region, which is involved in the recruitment and activation of co-activators [27].

### 2.4. Regulation Effect of AhR and ARNT on CYP6CY3

**Figure 6:**
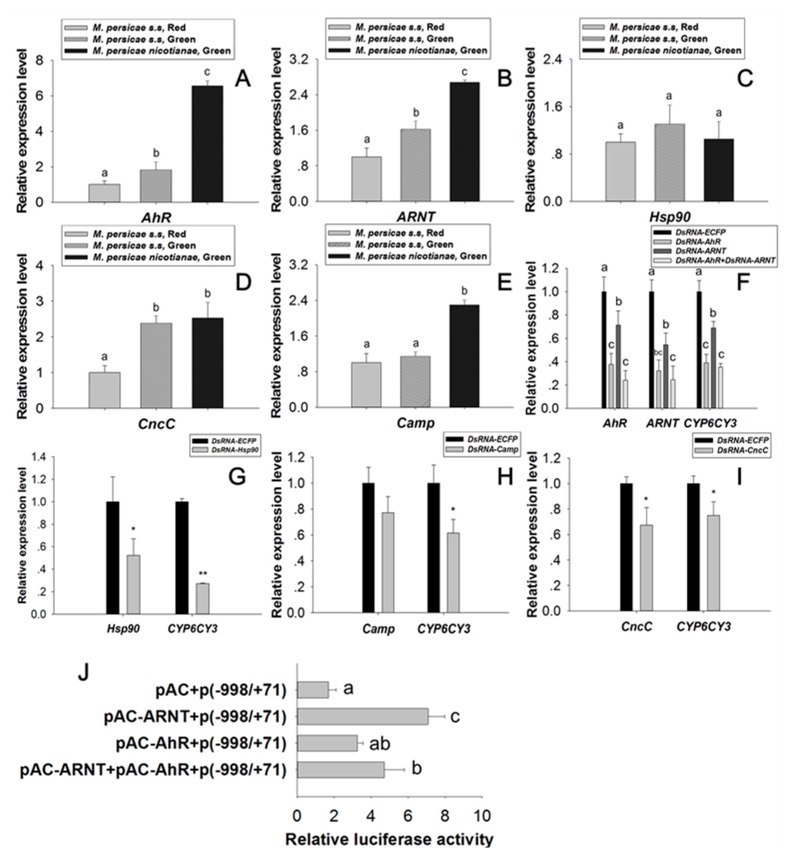
Functional characterization of *CYP6CY3* regulation by *AhR-ARNT*. (**A**–**E**) The relative *AhR*, *ARNT*, *Hsp90*, *CncC,* and *Camp* transcript levels in the three *M. persicae* races. (**F**–**I**) Orally mediated dsRNA knockdown efficacy (100 ng/μL of corresponding dsRNA) after 48 h and its effects on *CYP6CY3* transcriptional regulation. (**J**) The effect of *AhR* and *ARNT* overexpression on *CYP6CY3* promoter activity. The housekeeping genes *actin* and *para* were used as internal reference genes [24]. Different letters on the bars of the histogram indicate significant differences based on ANOVA followed by Tukey’s HSD multiple comparison test (*p* < 0.05). * Significant difference by Student’s *t*-test (*p* < 0.05). ** Significant difference by Student’s *t*-test (*p* < 0.01).

The expression levels of *AhR*, *ARNT*, and *Camp* (see sequences in Supplementary Data S3) in *M. persicae nicotianae* (Green) were significantly higher than those in *M. persicae sensu stricto* (Green) and *M. persicae sensu stricto* (Red) (Figure 6A,B,E). Orally delivered dsRNA mediated the RNAi knockdown of *AhR*, *ARNT,* and *Hsp90,* and dramatically reduced *CYP6CY3* transcription (the expression of *CYP6CY3* declined 60.97%, 31.20%, 64.76%, and 72.76% in the RNAi of *AhR*, *ARNT*, *AhR,* plus *ARNT*, and *Hsp90* treated aphids, respectively). The knockdown of *CncC* and *Camp* suppressed *CYP6CY3* expression (Figure 6F–I), whereas *CncC* overexpression did not increase *CYP6CY3* promoter activity (data not shown). Co-transfection of *AhR* and *ARNT* significantly elevated *CYP6CY3* promoter activity 1.92- and 4.18-fold, respectively (Figure 6J). The repression of *AhR* and *ARNT* expression also significantly down-regulated *CYP6CY3* expression in *A. gossypii* (Figure S4) [28].

### 2.5. Transcription Factor Modulation Impacts CYP6CY4 Expression

**Figure 7:**
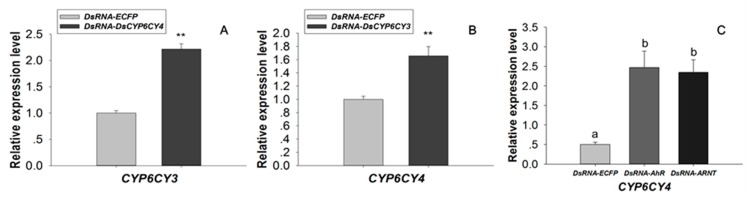
The effects of *AhR* and *ARNT* modulation on *CYP6CY4* expression in *M. persicae nicotianae*. (**A**,**B**) The relative *CYP6CY3* and *CYP6CY4* expression levels in *M. persicae nicotianae* orally treated with dsRNA (100 ng/μL dsRNA-*CYP6CY4* or dsRNA-*CYP6CY3*) after 48 h [23]. (**C**) Orally mediated dsRNA knockdown of *AhR* or *ARNT* (100 ng of *AhR* or *ARNT* dsRNA) impacts *CYP6CY4* transcriptional level. The housekeeping genes *actin* and *para* were used as internal reference genes [24]. Different letters on the bars of the histogram indicate significant differences based on ANOVA followed by Tukey’s HSD multiple comparison test (*p* < 0.05). ** Significant difference by Student’s *t*-test (*p* < 0.01).

A compensatory mechanism may exist between the *CYP6CY3* gene and its homologous *CYP6CY4* gene [23]. For example, *CYP6CY4* knockdown significantly increased the expression of *CYP6CY3* (2.22-fold) in treated aphids (Figure 7A). Similarly, the expression of *CYP6CY4* dramatically increased (1.66-fold) in the *CYP6CY3*-depleted aphids (Figure 7B) [23]. The orally delivered transcription factor dsRNA also had opposite effects on the transcription of *CYP6CY4* to those of *CYP6CY3*. The RNAi knockdown of *AhR* and *ARNT* dramatically reduced *CYP6CY3* levels (Figure 6F), but significantly increased *CYP6CY4* expression 4.93- and 4.69-fold in the dsRNA-*AhR* and dsRNA-*ARNT* fed aphid, respectively (Figure 7C).

## 3. Discussion

Herbivorous insects have evolved a complex of regulatory machinery and they have experienced substantial increases in P450 metabolic activity to address exposure to allelochemicals [8,9,10,11,21]. This response is essential for insects to counter the effects of toxins that are generated by host plants. Larval tolerance to gossypol in the cotton bollworm was reduced following *CYP6AE14* silencing with plant-mediated RNAi [29,30]. The overexpression of *CYP6CY3* in *M. persicae nicotianae* as compared to *M. persicae s.s* is partly due to gene amplification, which accounts for nicotine and neonicotinoid resistance [16,18]. The RNAi of *CYP6CY3* and *CYP6CY4* together is more effective for increasing nicotine toxicity to *M. persicae nicotianae* than RNAi of a single P450 gene (Figure 1). The regulatory cascades that are induced by exposure to plant toxins in insects remain largely unknown.

The motifs in the promoter region (*cis*-elements) directly upstream of the genes that are responsible for the binding of transcription factors, are likely candidate regions for controlling the transcription of tolerance-associated genes [31,32]. However, the identification of *cis*-regulatory elements poses a notable challenge. A *cis*-acting element, designated as the xenobiotic response element to the flavone (XRE-Fla), was characterized in *Helicoverpa zea* as a mediating xanthotoxin and the flavone-inducible expression of *CYP321A1* [33,34]. The CncC-Maf binding site that is responsive for xenobiotic-induced transcription in the promoter regions of the *CYP6BQ* and *CYP6A2* genes was identified in *Tribolium castaneum* and *Drosophila*, respectively [35,36]. A xenobiotic response element to the aryl hydrocarbon receptor (XRE-AhR) has been functionally characterized for both the *CYP6B1* and *CYP6B4* promoters in *Papilio polyxenes* and *P. glaucus* [19,22]. No such conserved element has been identified in the *CYP6CY3* promoter. Instead, tandem (AC)_n_ repeats were observed in the *CYP6CY3* promoter in *M. persicae* (Figure 2,). These conserved repeats were also found in the *CYPCY3* promoter in *A. pisum,* which shares high homology with the *M. persicae CYP6CY3* gene (Figure S1). Research showed that tandem repeats, combined with a variation of genetic and epigenetic characteristics, contribute to the evolutionary ability of organisms [37]. Repeat sequences are responsive elements in promoters that regulate gene expression by affecting local chromatin structure. [38]. Functional characterization indicated that the (AC)_n_ repeat is the responsive element for *CYP6CY3* transcription (Figure 3A–D). In humans, the (AC)_n_ repeat is a major contributor to transcriptional regulation, and hence to phenotypic variation [39]. For example, variation in the (AC)_n_ repeat number alone influenced the rates of transcription in the metalloproteinase-9 gene [40,41]. The (AC)_n_ repeats in the *CYP6CY3* promoters of *CYP6CY3* copies that were derived from *M. persicae nicotianae* were of significantly varied lengths. The (AC)_n_ repeat expansion influenced the transcriptional activity of its promoter until it reached up to 94 bp and was nicotine inducible (Figure 3E,F). Thus, (AC)_n_ repeat length (up to 94-bp) increased *CYP6CY3* promoter activity, which might subsequently elevate the nicotine tolerance ability of aphids.

Potential transcription factors, such as AhR, were identified by DNA pulldown combined with mass spectrometry and bioinformatics analysis (Supplementary Data S2). AhR belongs to the bHLH/PAS protein family. The bHLH domain participates in the binding of transcription factors to DNA and it promotes protein-protein interaction, and the PAS domains function as secondary dimerization domains, promoting interactions with another bHLH/PAS protein (i.e., ARNT) [42]. AhR is a ligand-activated transcription factor that mediates many of the responses to toxic chemicals. In the absence of the ligand, AhR is present in a cytosolic complex with heat shock protein 90 (Hsp90) [43], maintaining AhR in a quiescent (non-DNA binding) state. Upon binding with the ligand, the AhR complex translocates into the nucleus, and then dissociates from the molecular chaperone heat shock protein 90 (Hsp90) complex to form a heterodimer with ARNT. The alignment of *AhR* and *ARNT* of the *M. persicae nicotianae* with paralogs genes of *Drosophila* indicated that they share the highly conserved domain of bHLH and PAS (Figure 4 and Figure 5), which demonstrates that the *AhR* and *ARNT* of *M. persicae nicotianae* might have a similar function to *Drosophila*. The AhR/ARNT heterodimer binds the xenobiotic responsive elements (XRE) in the promoter, leading to the transcription of genes that encode xenobiotic metabolism enzymes, such as cytochrome P450 genes, which in mammals include *CYP1A1*, *CYP1A2*, and *CYP1B1* [44,45,46,47,48,49,50,51]. In our study, RNAi of *AhR* and *ARNT* dramatically down-regulated *CYP6CY3* expression in *M. persicae nicotianae* (Figure 6), and the co-transfection results further confirmed that the transcription of *CYP6CY3* is regulated by AhR-ARNT pathway in *M. persicae* (Figure 6). This process generates significantly elevated the *AhR* and *ARNT* expression in *M. persicae nicotianae* as compared to *M. persicae s.s.* and accounts for its tolerance to nicotine (Figure 6). A different promoter expresses the *CYP6CY3* homologous P450-*CYP6CY4* (Figure S3); its expression level dramatically increased when the *CYP6CY3* transcripts were knocked down, and the RNAi of *AhR* and *ARNT* had opposite effects on the transcription of *CYP6CY4* to those of *CYP6CY3* (Figure 7). However, *CYP6CY3* expression was significantly elevated when the *CYP6CY4* transcripts were repressed (Figure 7). This indicates that the *CYP6CY3* and *CYP6CY4* genes cooperatively cope with nicotine stress. These results, combined with the effects on nicotine toxicology (Figure 1), illustrate that *CYP6CY4* has a similar role in the response to nicotine tolerance and that the ability to shift the expression of specific genes might have driven the evolution of *M. persicae nicotianae’s* tolerance to nicotine stress.

The present study indicates that *CYP6CY3* are transcriptionally regulated by AhR and ARNT, and the significant up-regulation of *AhR* and *ARNT* expression further strengthen the transcription level of *CYP6CY3* in *M. persicae nicotianae*. This process, in combination with *CYP6CY3* and *CYP6CY4* switchover, conferred an adaptive evolution to nicotine. 

## 4. Materials and Methods

### 4.1. Insects and Cell Culture

Three *M. persicae* clones were established from a population originally collected in 2008 from a field in Jilin province, China. The *M. persicae sensu stricto* population (green/red morph) was sampled and reared on Chinese cabbage (*Brassica rapa chinensis*). *M. persicae nicotianae* (green morph) was collected and then reared on tobacco (*Nicotiana tabacum* L.) at 21–23 °C with a 16:8 h light:dark photoperiod.

Sf9 cells were routinely cultured in a SF-900 II serum-free medium (Invitrogen, Carlsbad, CA, USA) supplemented with 10% heat-inactivated fetal bovine serum (FBS, Hyclone-QB Perbio, Logan, UT), 50 U/mL penicillin, 50 µg/mL streptomycin, and 12 µg/mL gentamycin (Invitrogen) at 28 °C.

### 4.2. Quantitative RT-PCR and Data Analysis

Total RNA was extracted from the apterous adult aphids with TRIzol (Invitrogen, Carlsbad, CA, USA), according to the manufacturer’s instructions and treated with RNase-free DNase I (TaKaRa, Kyoto, Japan). cDNA was synthesized from the total RNA while using a PrimeScrip^TM^ First-Strand cDNA Synthesis kit (TaKaRa) with oligo (dT)_18_ as the primer. The housekeeping genes *actin* and *para,* which encode a voltage-gated sodium channel, were used as internal reference genes for *M. persicae nicotianae* [24].

Quantitative real-time PCR was performed on an ABI 7500 (Applied Biosystems) while using SYBR^®^ Premix Ex Taq™ II (Tli RNaseH Plus; TaKaRa). Gene-specific primers for real-time PCR (Table S1) were synthesized by Sangon Biotech Co., Ltd. (Shanghai, China). The thermal cycling protocol included an initial denaturation at 95 °C for 30 s, followed by 40 cycles of 95 °C for 5 s and 60 °C for 34 s. The fluorescence signal was measured at the end of each extension step at 60 °C. After the amplification, a dissociation step cycle at 95 °C for 15 s, 60 °C for 1 min., and 95 °C for 15 s was performed to confirm that only the specific products were amplified. Relative gene expression was calculated with the 2^−ΔΔ*C*T^ method [52]. The experiment was independently performed three times for each strain. Significant differences were analysed using the GraphPad InStat3 statistical software (GraphPad Software, 2000).

### 4.3. Cloning of CYP6CY3 5′ Flanking Sequences and Sequence Analyses

Genomic DNA from *M. persicae nicotianae* and *A. gossypii* was isolated from apterous adult aphids using the DNAzol kit (Takara). The GenomeWalker^TM^ Universal kit (Clontech, Palo Alto, CA, USA) was used to obtain the 5′-flanking promoter sequence of *CYP6CY3*. First, *M. persicae nicotianae* genomic DNA was digested with AfeI, EcoRV-HF, PvuII, SmaI, SnaBI, PmeI and StuI restriction enzymes. The genomic DNA of *A. gossypii* was digested by AfeI, PvuII, EcoRV-HF, SmaI, PsiI, PmeI, StuI and BstZ17I. Then, the digested DNA fragments were ligated into the genome walking adapters according to the manual’s instructions (see adapters and primers in Table S1). The resulting DNA fragments were used as templates for amplifying the *CYP6CY3* promoter sequence using two forward primers (AP1 and AP2) complementary to the adapter sequences, as well as *CYP6CY3*/*CYP6CY4* specific reverse primers (GSP1 and GSP2; 6CY4-GSP1 and 6CY4-GSP2) for *M. persicae* and two specific reverse primers (newGSP1 and newGSP2) for *A. gossypii*. The PCR protocol was performed according to the kit’s instructions. The bands were gel eluted using the TIANgel Midi Purification Kit (Tiangen) and were directly cloned into the pGEM-Teasy vector (Promega). The positive clones were selected for sequencing.

Searches for homologous sequences were performed while using BLASTN against the NCBI database (http://www.ncbi.nlm.nih.gov/). Promoter predictions for sequences with a score cut-off of 0.80 were identified with the BDGP database (http://www.fruitfly.org/seq_tools/promoter.html). Transcription factor binding sites were assessed by constructing matrices on the fly from TRANSFAC 4.0 sites while using TRANSFAC 4.0 software and the AliBaba 2.1 database (http://www.gene-regulation.com/pub/programs/alibaba2/index.html), with specific parameters (Pairsim to know sites value is 64, the match width in bp value is 10, the minimum number of sites is 5, the minimum match conservation value is 75%, the similarity of sequence to match value is 100%, and the factor class level is 4).

### 4.4. Construction of CYP6CY3 Promoter-pGL3 Constructs

PCR was employed to generate the following three types of *CYP6CY3* promoter reporter gene constructs: progressive 5′ deletion-pGL3 constructs, internal deletion-pGL3 constructs, and multiple base pair substitution-pGL3 constructs. The common template for progressive 5′ and 3′ deletion-pGL3 constructs was p(-2230/+71), which contained the 2301 bp 5′-flanking sequence from *CYP6CY3* that was obtained via genome walking. The obtained sequence was cloned into the pGL3 basic vector containing the first 29 bp of the *CYP6CY3* coding sequence, 42 bp of the 5′ UTR, and 2230 bp of the promoter sequence. Throughout this paper, we report the nucleotide position in the proximal promoter relative to the transcriptional start site (+1) with upstream positions being preceded by “-” and downstream positions preceded by “+”.

Progressive 5′ deletion fragments for 5′ deletion constructs, including p(-2230/+71), p(-998/+71), p(-903/+71), and p(-573/+71), were amplified while using the universal reverse primer 6CY3-XhoI and one of the forward primers 6CY3-2230-MluI, 6CY3-998-MluI, 6CY3-903-MluI, or 6CY3-573-MluI (see Table S1 for sequences). PCR was performed in a 50 µL mixture containing 10 µL 5 × PCR buffer, 1 µL 10 mM dNTP, 20 pmol primer pairs, 0.2 µg p(-2230/+71) template, and 2 U of LongAmp*Taq* DNA polymerase (New England Biolabs, NEB). The thermal cycling protocol was as follows: 94 °C for 5 min.; 25 cycles of 94 °C for 30 s, 55 °C for 30 s, 65 °C for 2 min.; and, a final extension at 65 °C for 10 min. The resulting band from each deletion fragment was gel eluted while using the TIANgel Midi Purification Kit (Tiangen, Beijing, China) and it was cloned into the XhoI and MluI sites of the pGL3-Basic vector (Promega).

Three internal deletion constructs (deleting the sequence from -221 to -138), including p(-998/+71)delAC, p(-903/+71)delAC and p(-573/+71)delAC, were generated using p(-998/+71), p(-903/+71), or p(-573/+71) as the template, respectively, and the ACdelF and ACdelR primer pairs. Two multiple base pair substitution mutation-pGL3 constructs, p(-998/+71)ACmut1 and p(-998/+71)ACmut2, were made while using p(-998/+71) as the template and two pairs of primers (MutF1 and Mut R1, MutF2 and MutR2; Table S1). PCR was performed in a 50 µL mixture containing 10 µL 5 × PCR buffer, 1 µL 10 mM dNTP, 20 pmol primer pairs, 0.2 µg template, and 2 U LongAmp*Taq* DNA polymerase (NEB). The thermal cycling protocol was as follows: 94 °C for 5 min.; 25 cycles of 94 °C for 30 s, 55 °C for 30 s, 65 °C for 6 min.; and, a final extension at 65 °C for 10 min. The resulting PCR band was purified with a TIANgel Midi Purification Kit (Tiangen, Beijing, China), end-blunted for 15 min. at 12 °C with T4 DNA polymerase (NEB), 5′-phosphorylated for 20 min. at 37 °C with T4 polynucleotide kinase (3‘phosphatase minus; NEB), and finally self-ligated overnight at 16 °C with T4 DNA ligase (NEB) to generate the constructs.

Based on the results, the DNA fragments from the promoters (-278/-28) containing the (AC)_n_ repeat region (shown in Figure 2) were amplified from single apterous adult aphid of *M. persicae nicotianae* and *M. persicae s.s* while using the primer pair Pulldown-F and Pulldown-R (Table S1). These DNA fragments containing 94-, 64-, 48-, or 32-bp AC repeats were cloned into the pGL3 basic vector while using MluI and XhoI restriction sites to construct p(-278/-28: 94AC), p(-278/-28: 64AC), p(-278/-28: 48AC), and p(-278/-28: 32AC), respectively.

### 4.5. Transient Transfection and Dual Luciferase Assay

The Sf9 cells were seeded onto a 24-well plate (4 × 10^5^ cells/well) and were then transiently co-transfected with *CYP6CY3* promoter-pGL3 luciferase reporter constructs (2 µg/well) and the internal *Renilla* luciferase control reporter plasmid phRL-TK (Promega; 0.2 µg/well) using the Cellfectin-II reagent (Invitrogen; 2 µL per well). After 48 h, the cells were harvested, and the resulting lysates were used to measure the *Renilla* and firefly luciferase activities on an FLx800TM fluorescence microplate reader (Biotek, Winooski, Vermont, USA). For nicotine induction, nicotine at a final concentration of 24.7 μM/L, or an equal volume of methanol (control), was added to the wells sixteen hours post-transfection. After 24 h induction, the cells were harvested, and the resulting lysates were used to measure the *renilla* and firefly luciferase activities. The relative firefly luciferase activity normalized against the *Renilla* luciferase activity reported for each construct represents the mean ± the standard error of three independent transfections. The induction folds reported are expressed as the ratio of the *CYP6CY3* promoter-pGL3 relative firefly activity to the basal relative firefly activity (pGL3 control). All of the experiments were repeated three times, and each figure shows one representative experiment. 

### 4.6. Isolation of Cis Element Binding Proteins, Protein Identification and Bioinformatics Analysis

The DNA fragments covering the (AC)_n_ repeat region (-221 to -138) were amplified while using p(-998/+71) as the template with the primer pair, PulldownF and PulldowR (Table S1). The DNA fragments were biotinylated with a Biotin 3′ End DNA Labeling Kit (Thermo), following the manufacturer’s instructions, and the biotinylated DNA fragments were immobilized with Dynabeads^®^ MyOne^™^ Streptavidin T1 (Invitrogen), according to the instructions. Nuclear proteins extracted from apterous *M. persicae nicotianae* adults while using an EpiQuik^™^ Nuclear Extraction Kit II (Epigentek, Farmingdale, NY, USA) were incubated with a biotin-DNA-Dynabeads complex for 30 min. at room temperature with gentle rotation. Next, the complexes were washed with PBS (pH 7.4) twice. Finally, the DNA binding proteins were eluted with 0.1% SDS for 8 min. at room temperature. The protein content was determined with the Bradford (1976) [53] method using bovine serum albumin as the standard. The protein sample was lyophilized and rehydrated in 30 μL 50 mM NH_4_HCO_3_ containing 50 ng trypsin. After overnight digestion at 37 °C, the peptides were extracted three times with 0.1% TFA in 60% ACN. The extracts were pooled together and then lyophilized. The protein sample was analysed with capillary high-performance liquid chromatography-mass spectral analysis (CE/HPLC-MS) by Aptbiotech Co., Ltd. (Shanghai, China). The identified proteins were searched against the NCBI and Uniprot databases and were then combined with the results that were obtained from the aphid database (http://www.aphidbase.com/).

### 4.7. Co-transfection of AhR, ARNT or CncC with CYP6CY3 Promoter-pGL3

Sf9 cells were seeded onto a 24-well plate (4 × 10^5^ cells/well) and were transiently co-transfected with p(-998/+71) (1 µg/well), pAC-*AhR*/pAC-*ARNT*/pAC-*CncC* (1 µg/well) and the internal *Renilla* luciferase control reporter plasmid phRL-TK (Promega; 0.2 µg/well) while using the Cellfectin-II reagent (Invitrogen; 2 µL per well). After 48 h, the cells were harvested, and the resulting lysates were used to measure the *Renilla* and firefly luciferase activities, as described above. 

### 4.8. Rearing on an Artificial Diet and dsRNA Feeding

We designed specific primers using the DNAMAN 6.0 software based on the *AhR*, *ARNT*, *Hsp90*, *Camp*, *CncC*, *CYP6CY3*, and *CYP6CY4* sequences (Supplementary Data S3) and the predicted possible interference sites from the online prediction software (http://www.dkfz.de/signaling/e-rnai3/). The gene fragments were amplified from cDNA and cloned into pGEM-T (Promega, USA). The purified plasmids served as templates for RNA synthesis while using the T7 RiboMAX™ Express RNAi System (Promega). *ECFP* dsRNA was used as the control and was synthesized under the same conditions as the primers (Table S1). The artificial diet and the rearing method that were used for this study were reported previously [23,54,55,56]. The diet was prepared in DEPC-treated water to ensure the absence of RNase activity. For the dsRNA feeding experiments, dsRNA was added into the artificial diet at a 100 ng/μL concentration. An artificial diet containing dsRNA-*ECFP* was used as a control. Sixty adult apterous *M. persicae nicotianae* were transferred onto the artificial diet device for rearing. To determine the effect of *AhR*, *ARNT*, *Hsp90, Camp*, and *CncC* knockdown on *CYP6CY3* expression, the aphids were fed an artificial diet containing dsRNA (100 ng/μL) for 48 h, and then the aphids were collected for RT-qPCR.

Sixty adult apterous aphids were transferred to the artificial diet containing nicotine (100 mg/L) mixed with dsRNA (final concentration 100 ng/μL), using the dsRNA-*ECFP* as the control, to assess the modulation effects of the gene expression on nicotine toxicology in *M. persicae nicotianae*. Three replicates were performed, and mortality was assessed after 48 h.

## Supplementary Materials

Supplementary materials can be found at https://www.mdpi.com/1422-0067/20/18/4521/s1.

## Abbreviations

P450	cytochrome P450 monooxygenase
EcRE	ecdysone response element
ARE	antioxidant response element
XRE	xenobiotic response element
XRE-xan	xenobiotic response element to xanthotoxin
AhR	aryl hydrocarbon receptor
ARNT	aryl hydrocarbon receptor nuclear translocator
XRE-Fla	xenobiotic response element to flavone
bHLH	basic helix-loop-helix
CncC Camp	cap ‘n’ collar/basic region leucine zipper (Cnc-bZIP) transcription factor; cyclic AMP-dependent transcription factor
PAS (PER-ARNT-SIM)	period (Per)-ARNT-single-minded (Sim)
Q-rich	Glutamine-rich
UTR	Untranslated Regions
TSS	transcription start site
CE/HPLC-MS	capillary high-performance liquid chromatography-mass spectral analysis
NCBI	The National Center for Biotechnology Information
SRA	Sequence Read Archive
Hsp90	heat shock protein 90
ECFP	Enhanced cyan fluorescent protein
dsRNA	double-stranded RNA
RNAi	RNA interference
qRT-PCR	quantitative RT-PCR
