# Supplementary material for: Transcription Factors AhR/ARNT Regulate the Expression of CYP6CY3 and CYP6CY4 Switch Conferring Nicotine Adaptation
Source: Int J Mol Sci. 2019 Sep 12;20(18):4521. doi: 10.3390/ijms20184521 (PMC6770377; doi:10.3390/ijms20184521)
Supplement: Supplementary file 1 [file ijms-20-04521-s001.zip › ijms-576626-supplementary data/Supplementary data 1.docx]

**Supplementary Data 1.**

**The expression of *CYP6CY3* and *CYP6CY4* in three subspecies**

| Gene name  (ID) | FPKM | | | | | |
| --- | --- | --- | --- | --- | --- | --- |
|  | *M. persicae nicotianae* (Green) | | *M. persicae sensu stricto* (Green) | | *M. persicae sensu stricto* (Red) | |
| *CYP6CY3* (c14664_g1_i2) | 481.42 | 228.22 | | | | 104.66 |
| *CYP6CY4* (c14664_g1_i1) | 173.22 | 71.09 | | 27.94 | | |

FPKM: Fragment per kilobases per million reads. The clean reads obtained in this study were submitted to the NCBI/SRA database (SRA experiment accession number: SRX1499035)[23].
