# Supplementary material for: Transcription Factors AhR/ARNT Regulate the Expression of CYP6CY3 and CYP6CY4 Switch Conferring Nicotine Adaptation
Source: Int J Mol Sci. 2019 Sep 12;20(18):4521. doi: 10.3390/ijms20184521 (PMC6770377; doi:10.3390/ijms20184521)
Supplement: Supplementary file 1 [file ijms-20-04521-s001.zip › ijms-576626-supplementary data/Supplementary data 3.docx]

1. The cloned complete open reading frame sequence of Cap‘n’ collar isoform C (CncC) of *Aphis gossypii*:

1 ATGTTGCGAA TTAAAAAAGG CTTTTCGGAG CAGTTGCTTC AGATGGCGTT GCTGTTAAGT

61 CTTATAGGTT ACGATCGTCT GTGGCCAACA TTTGGTATAT GGCCTTCGGC ACCTTCGGCC

121 GAAATAGAAG TAGGACCTTT GGATCGATGG GGTGACGATA GCTATTCAAA TGGCTTTGGT

181 CCATTAACTC AAATCCATCC CAAAAGTGTT GATCGGTATT TGGAGCGACA ATGGCTGTTA

241 GACGAATTAT TATCGTTGGG TAGATTTGGC GATCGATATC CATCAAATAT AGAGGCATAT

301 TTGCTTAATG TGGACGGTGA CGAGAACGGC CGAGACGATC AGCAGCAGCA AGACCCCGGT

361 GGTCAAGTTG TGAAAACTGA ACCTAAAGAA AATGATAGTG AAGACGAAGA TGTGGATAGA

421 GTATTTGACA CAGACATTGT TGAAGATGTT TTAATGCTTG CACCTGAGGA CGCTGAGCTC

481 GTAGAAGCTC TCTGGAAAGT CGATTTGGAT TATGGATCGT CCGGCGCCAC GTCCGATAGT

541 TACGGACCAT CTCCTTACAA CGTTAGCAGC GCTGGATCTT TCTTATCACC GGGTGATGAA

601 CCGCCATCGC GTAACCACAC TTTCAGCAAT CCTCCCAGTT CGTCTTATCC TCTTTGGCAC

661 ATTCCACCGT TGGATACCAA TTCGAGCGAC TTATCGCCAA GTTCCAGTTC ATTTTCGAAC

721 CGATTTGAAC GCACTCTCAA TTCTAATGGA AATTCCTGGG AGGTTGATAA TTTATTGGTG

781 GACCTCAAAA ATACAGGCGA AAGTCGTTCA AGACGAGTGT CCAGTGACAA TGATACCGAA

841 ACATCGGATT TGGATGCCGA TGAATCTGCC GAACACAAGT ATCAAGTACG ATCAAGTAGT

901 AACAACGCAT CTTTAAGCTT AACGCCAGGA TTTCGTGTGA AAGAATCTCC GTGGTACGAA

961 CCATCGTTTG AGTTTGACTT TGATGAATTC GAATATTTTG CAGACAATAT GATGCAGTGT

1021 TATTCTCAAC CACCGCCATC CTCTAGGCAT TTCCAAGGCA GGATGGGCTA CACGAGAACG

1081 GGCAACAGTG GAATGGACCA GCGAGGCTGG CAGGACTTGG CTACATGCAG CCCCATGTTA

1141 GCGTTCCCGG TCGAAGGACA ACACCACCAC CATCAGTCCC ATTATGGTCA GCATCCGTCC

1201 GGCGCACTCA GTTACCCGCC CGGACCCAGC ATGTATCACC ATCCTCATCA CCATCAACAT

1261 CACCACCAAC ACAACAACCA CAACAGCAAC AACAACACGG GAAACGTATT GTTGCAGAAC

1321 GTAAGCCTCT GCGCGCCTCC GCCGCCGCCA CCTCCGCCAG CGTCTACGTC CATTGACATG

1381 GGTGGAGGCC ACGCGACCAC GTCATCTTCG TCGTCGTCCC TATCACACTA TCATCACCAC

1441 TCTTCATCGA TCGGTGTTTC GACCAACTGC AATCTGAGTT CGGCCGTCGC CACTTCGCTT

1501 CACTTACCCG GCAGCTCATC CGAACAGCCT CCTGCGTTCA AGACAGAACC TCATCAACAT

1561 GAAATGATGT TTCCCTATCA GAATCACAAT AATGAAATGG CGCCTTCATC GGATGGACTT

1621 TTGTCGTCGA TATTAAACGA TGACGATTTG CAGCTTATGG ACCCGTCGAT GGGTACAGAC

1681 GGTGGTATGT ACCCGGTGAG GATGATGGAC ACAGCTAGCA GCTCAGTGCC GTGCAACAAC

1741 AATATAATGA ACACCGCAAC CATGGTACAA AACGGTAACG CGGGTGGTGG TGACTCGGAC

1801 AGTGCCGTGA GTTCGATGGG CTCAGAACGC GTGCCCTCGT TGTCTTCGGA CACCGAATGG

1861 ATGGAAACAA ATTCAGACAG CGGACACAAT GACGGGTACC CGTCGCATTC CAACCAAGAA

1921 TATGGAAAAA TGAGGTGGTT GGACAACTAT CTGTATTCCG GAGGTCGTGG TCACTCGGCT

1981 GCTCCACAAA AGAAATATCA GTTGTATGGT AGGCGCCTAA CAGATTCTTT ATCAGCGACC

2041 CCGAACGCCA CTGAAACGTC AAGGCCTAAT CACGTAGGTC TAGTGCCCGA TTCATTGAAC

2101 CTGCCGTATG ATATGTCTGT CAGAGAAGGC ATCCCAGTGC CCGGCTCTTC ACAGTTTAAC

2161 CGGCACATGG CTGGTCCCAG GACACCACGT AACAAGTTCA AGAATAGTAA GAGGAGCGAG

2221 GATGAACAAC TTACAAGAGA CGAAAAGAGG GCCAGAAATC TGAATATTCC TATTAGCGTC

2281 GACGACATAA TCAATCTGCC CATGGACGAA TTTAATGAAA GGTTGTCCAA ATACGATCTA

2341 TCTGAAAGTC AATTAACATT AATTAGAGAT ATTAGGCGTC GAGGAAAAAA CAAGGTGGCT

2401 GCTCAAAATT GCCGGAAACG TAAGCTGGAT CAAATCCTCA GCTTAGCCGA TGAAGTAAAA

2461 CAAATGAAAG ACCGAAAGTT CAATCTGTTA CAAGAACGTG ATTACTTAAT GACCGAGCGT

2521 GTCAGAGTCA AAACCAAATA TGATCTACTT TATAACCATA TTTTTGGAAG TTTAAGAGAT

2581 CCTGACGGAA ATCCGTACTC ACCATACACT CATACGTTGC GTGAGCAACC CGACGGCAGT

2641 GTCAGTATTG TACCCAGAAA TAATGGTACC CAAACCGACC GGAACCCTCG GTCCAAGCAA

2701 CACAAGGACA AGTAA

1. The cloned complete open reading frame sequence of aryl hydrocarbon receptor nuclear translocator (ARNT) of *Myzus persicae*:

1 TAGTGTTTGT GTGTGTGTTA CCGTCTCATC ACTCGCAGTT CGAGCTCAGT GAAATAGGAA

61 TTTCGTTTTT TAAATTATAT ATTTTAAATA TATAATATTT TGTCGGTGTT GTTCGTTTCG

121 ATAATTCCGT GGGTTTATTT TTATGGTGTT TCGCGACATA TATGTCGTCT CGGCGACGGC

181 TTCCGGACGT GAAAACGTCG CGACCGCGGT GGTGCTATGA GCCACTGTTG GCGGCTACGA

241 CTACCGCTGT AGCAGCAGCC GGATGTACGG ATACGCGGGT CCGCCCCATT ACGGGGCCAT

301 CGAGTCCTCC ACTGGAGACC CGGGCGGCTA TCTGCAGCCT GGTCCCATAC CCATGGCCAT

361 GGTCATGCCT ATGCCGGCCG TCTGTGAACC ACCACCGCCA CCGCCGCTTC AAATGCCCGA

421 TGAGGCGATA CAGAAACGGC GGAGATCCGA TGAAGAGGAT TCTAGTCAGA AATATTCTAG

481 AATGGAAGAT GATAATATCC AAGACAAAGA AAGATTTGCC AGTAGGGAGA ATCATTGCGA

541 AATTGAGAGG CGGCGGCGGA ACAAGATGAC AGCGTACATC ACGGAACTCT CGGACATGGT

601 GCCGGCATGT CAGTCCCTAG CTCGGAAACC GGACAAACTC ACCATACTCC GAATGGCTGT

661 TAACCATATG AAAAGTCTCA GGGGTACAGG TAACACAAAC AATGATGGCA CCTACAAACC

721 TAGCTTTCTA ACGGATCAAG AGCTTAAACA TTTAATATTG GAAGCAGCTG ATGGTTTTCT

781 ATTTGTTGTT ACATGTGATA CTGGTAGAGT TATATACGTT TCCGACTCTG TTGCACCAGT

841 ATTAAATTAC TCACAAAATG ACTGGTTAGG GACAAGTATG TTTGATCATT TACATCCCGA

901 AGACGTAGAA AAAGTACGTG AACAACTATC TACACAAGAA CCACAAAATT CAGGGCGTAT

961 ATTAGATTTG AAAACTGGTA CAGTGAAAAA AGAGGGTCAT CAATCTTCAA TGAGACTGTG

1021 CATGGGTTCT CGACGTGGGT TCATTTGTCG AATGAAGATC GGTAATAGTG GTGGTATGAT

1081 GTCCAGTATA TCGGGACATA ATCTACACCA ACGTCTTAAA CAGCGTAATA CATTAGGACC

1141 AACTCGAGAT GGTAACGAAT TTGCGGTCAT CCACTGCACT GGCTACATAA AGAACTGGCC

1201 TCCATCTGGT GTTCAAATTG AGAGAGCTGT TGAAGAAGAT GGTACTCATT GTTGTTTAGT

1261 TGCTATTGGT CGGTTGCAAG TTACTTCCAC TCCAAACACT ACTGATTTAA CCGGGTCTAA

1321 TAGTAATGCT GAATTCATTT CTAGACATTC TATGGATGGT AAATTTACAT TTGTTGATCA

1381 GCGTGTTACA CATATACTTG GCTATAAGCC ACAAGACCTT TTAAGCAAAA CATGTTATGA

1441 GTTCTTTCAT CCTGAAGACC AAACTCATAT GAAAGAAAGT TTTGAACAAG TTCTTAAAAT

1501 GAAAGGCCAG ATGATGTCTG TGATGTATCG GTTTCGAGGT AAAAATCATG ACTGGATTTG

1561 GCTAAGAACA AATGCATTTG CATTTTTAAA TCCTTACACT GATGATATTG AGTATATTGT

1621 ATGCAACAAT TCAACGGCTA AATCCTCGAT GCACTCCCCA TCTGAAAGTG GAGTAAATAT

1681 CAGTAACCCA CCAACTGAAC CTGTTTATCA ACAACAGGCA CCTGGATTAG ATTATACAGT

1741 TCAAAGAAGG GATCATGTAG CAGCACCACC ATATCAAGCT CATGCAATGA TTACTGCTGC

1801 TCCGACCGCC AGTCAACATA TGATACCCAA CAACACAGCG CAAAGACCAA ACAGTGCTCA

1861 AAATGTATTT AATACATATG AAACGAATGC ATCACCCATT AGCTATCATT CGCCCAATCA

1921 AAGTACTCAA AGTCAAGTTC AATCCCCATT AATTAATCGC CTTACTAAGT CAAGTCCAAC

1981 ACCTGCACAA ACAGCATGGA CATTGCGCCA GCCGGTCACT GAAGGTTACC AATATAATCA

2041 AGAAATGAGT CCATCTCGTT CACCATCAGG TCCAATATAC ACTCAATTGA GTGGTGGTGC

2101 AAGACAAACA TCTTACCACA ATCCATCACC AGCAACTGCG TCTCCAGGAA TGTGGGGTTG

2161 GCAAGCTGGA GCAACTGGGG CCACAGCTGT GGCCAACAAT GTACCACCAC ATCCGCATAG

2221 TGGCCATCCA CAAGAACTGT CTGATATGAT GATGCGGATG CTTGATCAGA GTGCAGCAGC

2281 TGCTTCATTT GAAGACCTTA ACATGTTCAA CACAAATTTT GAATGAGCTA ATAATTTTTA

2341 TATTATATAC CTATGTTGAA CAAATGTATT AAGAACTAAA TGAATGCAGT TGTCACAAAT

2401 TACCTGAGTG TATGCTATAA ACTAAATTTG TTTAATATCT GCCTGTAAGG TATTATCAAC

2461 AC

1. The cloned partial open reading frame sequence of aryl hydrocarbon receptor protein (AhR) of *Myzus persicae*:

1 CTGACATCGC AGGTTGCTCG AGGCGCCCCT AAAACGGAGT ACGTCACGGG GACGGGGCCA

61 TGCGGCGCCC CCCCAGCCAC ATACCATCAG CCACTGATCG AACACCATCA GAACCACGTG

121 CAACACCATC AGCTGGTGGC CGCGTCCAAC GGACAGTTGC GCGGTCAGTC CAGCGCCGCC

181 GCGATGGCCG TGGCCGCGGT CACAGCACCG CCAGTCCAGT CTGTCAACCC GCTGAGCACC

241 GTCTACGCGA CCAAGCGGCG ACGGCGCAAC GGCAAGAGTG TGAAGACGAC GCCCAAAGAC

301 GGGGGCGTGG GAAAGAGTAA CCCAAGCAAA CGGCACCGCG AACGGTTGAA CGCCGAGTTG

361 GACACGCTGG CTAACCTGTT GCCGTTCGAA CACAATATCC TATCTAAGTT GGACCGGCTG

421 TCCATACTCC GGTTGTCTGT CAGCTACCTG CGTACCAAGA GCTATTTCCA AGTTACTATG

481 CACAAGTCCA AAGAAGAACA AGCCAATGTT ACCAGCTCCC AGTACCAGCG TTCCAGGACG

541 TGCCCGGAGC CACATCATTT GTACTTGGAC GGAGATATGT TCCTACAAGC ATTAAACGGA

601 TTTTTGGTGA TGCTCACTTG TGACGGGGAA GTTTTCTTTG CTACACATAC CATAGAAAAT

661 TACCTTGGCT TCCACCAGTC CGACATCGTT CACCAGTCAG TCTACGAACT AGTGCATTCT

721 GAGGACCGAG AAGAGTTGCA GAGACAGCTC ATGTGGAATT CGGCCATACC CACGGAACCG

781 GGGTCGCCGT CTCCGTCCAT CACATTGCAC GAGGCCTTGC ACCCGGATAA CGGCCGTCTG

841 TTGCAGCGTA GTTTCACCAT CCGTTTCCGG TGTCTGTTGG ACAACACTTC CGGTTTTTTG

901 AGACTGGACG TCCGCGGACG GATCAAAGTA ATTCATGGCC AAAACCGCAA GTCAGACGAA

961 CCACCACCAT TGGGCCTTTT CGCGCTATGC ACGCCCTTCG GCCCGCCGTC TTTACTTGAA

1021 ATACCGCACA AGGAAGTCAT GTTTAAGAGT AAACATAAGC TAGATCTCAG CCTAGTGTCT

1081 ATGGACCAAA GAGGCAAGTT ATTATTAGGT TACTGCGATT CCGAACTCGC TAATATGAGA

1141 GGATACGACT TAGTCCATTA TGATGATTTA GCGTACGTGG CGAGCGCCCA TCAAGAACTG

1201 TTGAAGACTG GAGCATCAGG AATGATTGCC TATAGGGTGC AGACCAAACC GGGCACGTGG

1261 CAATGGCTAC AGACTAGTTC CAGACTGGTT TATAAAAACT CGAAACCTGA CTTTGTGATT

1321 GGCACGCACA GACCTTTAAT GGAAGAAGAA GGCCGCGATC TGTTGGGCAA GCGGACCATG

1381 GACTTCAAGG TCAGTTACTT GGACGCGGGA CTGACGAATA GCTACTTCAG CGACTCGGAA

1441 CAACTGTCCG GCACGTTGAC GACGTCCACC ACCGCGGCGT CCACCGCCGG TGCCGGCACA

1501 ACCGGGTCGG CCGGTCAAAC GCAACCGTCG CAGCCTAGAC GCCGGTACAA AACGCACCTA

1561 CGCGACTTTC TGTCAACGTG CCGGACGAAG CGGAAACTGT CGGCGAGCAC CGCGACACCG

1621 GCACCGACGG TGGCCGCCAC CGTGGCCGTC GATTATCCGA CGGCTGTCCA GGCGCCTTCG

1681 GTGCCCGCCG CCCCGCCGCC GACCGCGGAC GTCCTTTACA CAAACCTAAA CACGGCGGCG

1741 CTGTACTCGG CCGGACCGTA CAACGGGGCA GTAGTCGCTG ATAACGGTGG ATACCACCAG

1801 ACGAATTTCC ATCAGACCCT CTACGATACT CGAATACCGT ACCTGACGGC CACTGACAAC

1861 CTGTTCCAGT ATAGGCCTTT GGGCAACTAT TACACTGAAT ACCACACACC GGCCACTACA

1921 CCATACATGG GCAATGGGTT TTTGGACATA AGTCCCAGGG GACCAGTGCA GTGTGGTCTG

1981 CCCACATACG ATCAACTGAC CACGGTAACG ACGGCGACTG TCGGTGGCAG AGAATGCGGC

2041 GACAAGCTGT ACGGATCGCC ACCAGTAGTG TCGATGGATC ATCACCAACA ACAGCAGCAG

2101 CACCTACACC ACCATCACCA CCACCACCAA CAGCAACAGC AGCAACAGCA ACAACAGAGC

2161 GGCGAAGACA ACATAGGAAA ATGTCATATG GACACCTCCA CGGCCGCCGC CGCCGTCACC

2221 TGGGCGG

1. PREDICTED: hsp90 co-chaperone of *Myzus persicae*:

1 TCCAGAAATC GCTAATCAGA ATAATATTGT GCATTGTGCT TGTACCCTTT GCGCAGTTAA

61 TACTGTTTAG AAGTTTTACT ATTTTCTATT CTCGATAGTG AAGTATGTTC AATGATTAAA

121 ATAGTACAGC ATATTACAAA ATAATCTATA CAATTTGTTC TTTGATAGTT TGATACAGTT

181 AATAGTAAAA AATTATCTAT AATGGTTGAT TATAGCAAGT GGAAGAATAT TGAAGTATCC

241 GACGATGAAG ATGAAACCCA TCCTAACATT GATACACCAT CCTTATTTCG CTGGAGGCAT

301 CAGGCTCGTA TTGAACGTAT GGAGGAAATA AAACGTGAAC AACAAGAATT GGAAATCAAG

361 AAAAAAACTT TTAAAGAAAA GTATGAAGAA ACTAAAAATC AGCTTTTGTC AGCTGAACAA

421 GAAGGAAAAA ATAAAAAAGA ACTCGAGGAA GCACTAAGTG CTTTAAGTGT TGAAGAAGAA

481 GAATTAAAAA AAAGAGAAGA AGAGTTCAAA GTTAAAGAAA AGGTAATGCC ATGGAATGTT

541 GATACTATTA GTAAACCAGG TTTTACAAAG ACGATTGTGA ATACTCCCAA GCCTCCACCA

601 ACTGAAGAAA ATTTGACTGA AGAAGATAAA GCTAAGCGAT TAGAAACATT TATTAATGAA

661 AATAAAAGTA AATTGAAGGT ATTTGGTATG TTCAGAAAAT ACAAAGACAG TCAGGAATAT

721 TTACAAAAAA ATCCTCAACT TGTTTGTGAA GATACCGCAA ATTATTTGGT TATTTGGTGT

781 ATTGATTTAC AAATGGAAGG TAAATCAGAT CTAATGGAAC ATGTAGCACA TCAAACGATT

841 TGTATGCAAT ATATTTTGGA ATTATCTAGA CAACTTAATA TTGATCCAAG AGCATGTGTA

901 CCTTCATTTT TTTCAAGAAT ACAATTAGCT GAGAAACAGT ACAAAGATTC TTTCGATGAA

961 GAGCTTAATA TGTTTAAAGA TCGCATCCGA AAAAGGGCTG AGGAAAAACT GCGTATAGCT

1021 CAAGCTGAAA TTGAAGAAGA AGAACGAAAA GCAAGATTAG GACCAGGTGG TCTTGATCCA

1081 GTAGAAGTTT TTGAGAGTCT TCCAGATGAG TTGAAAAAAT GTTTTGAATC ACAAGATATC

1141 CAGTTATTGC AAGATACAAT TAAAAACATG AACCAAGAAG ATGCAACTTA TTATATGAAG

1201 CGCTGTGTGG ATTCTGGATT ATGGGTACCT GACGCCAATA AAGATAAGAG CAGTGCTGAA

1261 GATGATGATA AACCACAAGA AGAAAATATT TATTCTGAGA TAAACAGTTC ATAAAATAAT

1321 CTTATATTAT CCTATACATT GATTAACTTA TAATTATTAG ATTATAACAT TTTTTCTGGT

1381 TCTAAATTTA TCTGTTTACA TACAAGACAT GAAATACTTT TGGTTGCATT ATTATAGTAA

1441 AACTAATTTT TTTTTATAAT CAAATTTTGT TTGTTAAGTA TTTATAATGA TAATGATAAT

1501 AATATTTATA ATATTATTTA AAATAATATT TGTTTATCGT GATTGTACAT AATTATCAAT

1561 TTTTTTCATT TTTTTCCGTA TTACTGCTTT TATCACATTC TTTTTGTAAA AATGGTAAAG

1621 GTATAGGTAT AGGAATAGGT ATTGGTATTG GTAACATAAT AGGATATGGT ACTAATATCA

1681 TAGGTGGTGG TAGTAGCGAT GGTGGCAAGT TCAATGGGTA AAGTGGCGAA GGTGTTGGAT

1741 CAGCATTTGT TGGTATGCTG CTACTTGTTG ACTGAGACTG TGTGAATGCT GGTGACCTAC

1801 ATATCGGTGG TGTACAGTAA ACTGGCGCAT GTGGTGGAAA TTTTGGGTAT ACAGGTGGTA

1861 CAGTATCTTC GACTGTGGTT CTTTTACGTT TTCGGGTTTG ACGGTCAAAC AGTTTACTGG

1921 ATATGCGTAG TTGAGGAACT GCCGGTCCAG GCAGTGGAGG GGAATGAGGC CTTTGAGGAG

1981 ATGGTGACCG GTCTGGAAGT GGCGGTGAAT GACAATCGCG TAGCCATAGA TCGGGTGTAA

2041 TGAGCGGCAA GTTCGGCTCC AGGGTTTTAC AAAGGTTTTC TGGCAGCGAC GTGTTTTGTA

2101 GGTGAGGGTG TAACTCTAGC TGGGCTTGAG TTTCTTTGCA GAAGATGTTC ATCTTATACT

2161 GGTTCAGACA TTTATCGGTT TGTCACCTGC AGAATTGTAG CTGATGATCG CCGTCTTGGA

2221 AATCAACACA GTTAACTGTG TGTCTCAGAT GTCTACACCA GTCGCATGTT TTATTTCGTT

2281 TGAAATTTGC CCTTCGGTAG TGTGAGAAAC ATTGTTCGGA ACAAAATACT TTTTCGTTAT

2341 CCGAGTCTGC CGTGCGGAAT GTAAACGCTT TGGCTATTAG CGTTTTGTCA CACCATCCGC

2401 ATGTCATTTG CTGTACTGGT GAGTTCGACG TTGTTGGCGA TTGTGGACTA TTGTATTCGT

2461 CTGACGAAGT TTCTGGTTCC GTGCTTTTTG AGCGGCCTCT ATATTTTTCT CTAAGCTTAA

2521 CTAAATTCAT ACTTTGCAGT GAACAATCTT TGGAATTAAT TTTTTCATAT CCGTACCATC

2581 CGAGCATCTC GTTCATTGTG GTTTTTACGT ACTCTTTGAT CTCTTCGTCC GGAGTTTCGT

2641 TTTTCACCAA CCCTTTGGAG TCCGAATCCT TTCGAACGTT TCTGTTCATC TCTCACAATA

2701 ATGCTATAAT ATCAATTCGG TCGATCGATT ATTTTATTAC GACTGTGCAG GTATAGACTA

2761 GCGCGAGTCG AGGCGCCGTA ATCTCTCTCT ATGTATATAT TCGTACACGC GCACTATACT

2821 GACAAACTCT TAAACACGAA AATATTTACA TTACATTTAC ATACACACGA AATATTAATG

2881 TTTAATGGAT TCGATTAAAA AATATAATAT AATTGCATAA AGTCGTTTTT GAAAATGTGC

2941 GGTTTTGGAC GACTGCGGCG GCGTTTATAT CGAAGGACGG CGTGTGACGG GCACCGAGGG

3001 ACGAGACTCA CTCGGCAGTG CGAAAACGCG CCACGGCCGT CGTAGCTTAT AACGACGTGA

3061 TGGTCGTACA CGATATAATG TGACCGTCGC CGCGCGCTCG ACAGCGTATA TAATATAATA

3121 TAATAACGTC GAGAAACAGG CGCGCGTCGC GTGG

1. c14664_g1_i1: PREDICTED *CYP6CY4* of *Myzus persicae*:

1 CTTTAACGCT TATGGCATAA TAACAATTTC GGTTGAAAAT TGTATTGTAC AATTAAATAG

61 TTAAAGTAAA TTATCCAATT AAAGTGCCAA TCATTATACG TAGGTATCAA TATTAAAATT

121 AATGTTTTAC ATATTATTAT GGGCTGACCT ACTACAGCTG TGGTTCGATG GACAATAACA

181 CATTACCGTG TGAAAAACAG GAAACCCCCT CCGAACACGG AGGTCATAGT TATAAATATA

241 AAAAATATAT ATACTCTCGA AAACATCCAA CGGAATGTTA TCTTTGTTAC ATTAGATCGT

301 ATACAGCATT AGTGTCCACT TTAAAGGATT GCGGTGTTGA CTTCTGTCGG TAAGTTGTTT

361 GTCGTATTCC GTGCAAACGA TCTCCGGCCA TGTTCACCAC CGATTGGTGG ATAAATGTCG

421 TTACGGCATG CACGATAATA GTGACGATCG TCTATTATTT CTGCGTGTCG ACCTTTCAGA

481 AATGGGAAAA GCTCAACGTG CCGTACATAA AACCGATCCC GTTGTTTGGG AACTTTTTGA

541 ACATAGCCTT GGGCAAGGAC CATCCGCTGG AATTTTACAA CAAAATCTAT AACGAGTTCG

601 AGGGTCGCAA ATATGGAGGA CTGTTCCAGA TGAGAACGCC TTATTTAATG GTCCGCGATC

661 CCGAAATAAT CAACGACGTG ATGATAAAAG ACTTCTCGTC GTTCCCCGAC CGCGGAATTT

721 ACTCGGATTT CGCGGTCAAC CCGTTGTCGA ACAACCTGTT TTTCATGGAA AATCCTCAAT

781 GGAAAACTAT AAGAAACAAA TTGACCCCCG CTTTCACGTC GGGAAAGCTC AAGACAATGT

841 ACGATCAGAT CAAAGAGTGC GGAGACGTAT TGATGAAAAA CGTCGATATC AAATTAAATG

901 AAAACAACAA CGAAATAGAA ATAAGGGACA TCATGGGAAA GTATTCGACT GACGTCATCG

961 GCACTTGCGT TTTTGGCCTC AAGTTGAACG CCATAACGGA TGACGAATCC CTATTTCGTA

1021 AGTACGGCAA ATCGATATTC ACACCTTCAA TGAGAATGCT TTTCAGAGAA TTGTGTTTGA

1081 TGATTACTCC TGCACTTTTG AAAGTCGTAA GAGTGAAAGA TTTTCCAACG GATGCGACTG

1141 ACTTCTTTCA CTCGGCGTTT AAAGAAACGA TAGCGTATAG ACTTGAAAAT AAAATAGTCA

1201 GAAATGACTT CGTTAACTGT TTAATGCAAG CAAGAAATGA TTTAGTGTTG AATAAAGATT

1261 TACCTAAACA TGAAAAATTT ACTGAATCGC AAATCGTTGC AAATGCTTTC GTAATGTTTG

1321 CTGCTGGATT TGAAACTATA TCCACTACTA TAAGTTTTTG TTTATATGAA TTAGCATTAA

1381 ATAAATCTAT ACAAGACAGA GTACGCCAAG AGATTCAACT AAAACTGTCC AAAAATGACG

1441 GACAAATTAA CCATGATTTT TTGATGGATC TTAATTACTT GGATATGGTT ATAGCAGAAA

1501 CTCTTCGTAA GTATCCTACT TTGGTTGCTT TGTTCAGAAA AGCATCACAA ACATATCAAA

1561 TACCCAACGA TTCATTAACT ATAGAAAAGG GCCAAAAAAT AATAATTCCA GTTTATGCGA

1621 TGCATTATGA TCCTAAGTAT TATAAAGACC CGGAAAAGTT TATTCCTGAA AGATTTTCGA

1681 CTGAAGAAAA AGCAAAACGA CCAAATGGTA TTTATCTTCC ATTTGGCGAT GGCCCTCGAA

1741 TGTGTATAGG AAAACGTTTC GCGGAGATGG AAATGAAATT GGCTTTTGTT GAAATATTAA

1801 CCAAATTTGA AGTATTCCCA TGTGGCAAAA CAGAAATACC TCTAAAATAT TCGAATAAAG

1861 TTTTAACATT GATGCCGAAA CATGGAATTT GGCTAAGATT TAAAAGAACT GAATGACCAA

1921 AGATTAAAAT TTATAGACTA AAAGAAACAT TGAAGTTTTA ATGTGACTGC CAACCGGAAA

1981 AAAATGAACG ATTTTTATTT TATTTGTTGT ATCGAGATAT TTTCAACATT TCCCATTATC

2041 TTATATCTTA ATATTAAATG AAAGGGAATC AAATAAACAT ATAAAATACA TTATATATGA

2101 GTACCTATAT GAATTGATAA TAAGTATGTC ATGAATTATT TTTATAATGT TGTATTTGAG

2161 GTAGGCATTT ATTTTTGTAG TCGTATATAA TGATTTATAA CTAATACCAA ACATTAATAT

2221 AGTTTACTCA ATGTCCAAAA ATGAGTGTCC TGTGCTGATA CAAACCTTTG TTTTTCA

1. c14664_g1_i2: PREDICTED *CYP6CY3* of *Myzus persicae*:

1 ACACACACAC ACACACACAC ACACGAGCAA ATACCTCATA AATGTGTGGA CACCAAATGC

61 GAAGTGATTC AGATAACCCC ACCAAATTAG ACGGTGCTGG TACCTTATAA TTCTCTTTAT

121 AATATTATTA CTCTCTGCTG TCTACCAAGG AACAGTGTCG ACTTGAGTGT GTACTGTGTA

181 TGGCGTATAG TTCATAATTG TTTATAATAC CGACCAGGCC ATGAATCTAT CATCGTCAAC

241 CACCGACTGG TGGATTTATA TCGCCTCGGC GTGTTTAGTC GGGGTGACGA TCATCTATTA

301 CTTTTGCATT TCAACGTTCA GTAAATGGGA AAAACTCAAC GTGCCCTACA TCAGGCCGAT

361 TCCGTTGTTC GGAAACTTTG TGAGAGTAGC TTTGTCAAAA GACCACCCTT TGGAGTTTTA

421 CAACAAAATC TACTACAAGT TTGCTGGTCT AAAATACGGA GGACTGTTCC AGATGAGGAC

481 ACCGTATTTG ATGATTCGTG ATCCAGAAAT AATCAACAAC GTGCTAATAA AAGACTTCTC

541 GTCTTTCCCA GACCGTGGTA TTTACTCGGA TTTAGCGGCG AATCCATTGT CGGACAACTT

601 GTTCTTCATG GAAAATCCCC GATGGAAAAC AATAAGAAAC AAATTGACCC CCGCTTTCAC

661 GTCGGGAAAG CTCAAGACGA TGTACGATCA GATCAAAGAG TGTGGAGACG TATTGATGAC

721 AAACATCGAC AAGTGTTTAA GGGGGGGAAA CGAAGAAATA GAAGTAAGAG ACATCATGGG

781 GAAGTATTCG ACCGACGTCA TCGGCACTTG CGCTTTCGGG CTCAAGCTGA ACTCCATAAG

841 CGATGATGAA TCCCCATTTC GCAAGTACGG AAAATCGATA TTCATACCTT CACTAAGAAC

901 TCTTTTCAGG GAGCTGTGCC TGATGGTGAC CCCCTCACTT TTGAAAGTTG TAAGGGTGAA

961 AGATTTTCCA ACGGATGCGA CTGACTTCTT TCACTCGGCG TTTAAAGAAA CGATAGCGTA

1021 TAGACTTGAA AATAAAATAG TCAGAAATGA CTTCGTTAAT TGTTTAATGC AGGCAAGAAA

1081 TGAATTGACT TTGAATGCAA ATTTACCCAA AGAAGAAAAA TTTTCCGAAT CACAAATTGT

1141 AGCAAATGCT TTTGTAATGT TTGCTGCTGG GTTCGAAACA ACATCAACTA CTTTAAGTTA

1201 CATCTTATAT GAATTAGCGT TGAATACGTC TATTCAGGAC AAAGTACGTC AAGAGTTTCA

1261 GTTGAAATTA TCCAATAGTG ATGGACAAAT TGACAACGAA TTTTTGATGA GTCTTAATTA

1321 CATGGATATG GTTATTGCGG AAACCCTCCG TAAGTATCCT CCTTTAATTG CTTTATTCAG

1381 AAAAGCATCA CAAACATACC GTTTACCTGA CAACCTAATA CTGGAAAAAG GCCAAAAAAT

1441 AGTAATTCCA ATTTACTCAC TCCATTTCGA TGATAAATAT TTCGAGGATC CTCAAAAATT

1501 CGATCCTGAA AGATTTTCAC CCGAAAACAA AGATAAACGT CCTAATGGTG TTTATCTTCC

1561 ATTTGGTGAT GGACCTAGAA TGTGTATAGG AAAACGTTTT GCTGAGATGG AAATGAGATT

1621 GGCTTTACTC GAAATGTTGA GCAAATTTGA AGTCCTACCA TGTGAAAAAA CAGAAGTTCC

1681 TCTAAAATAT TCTAACAAAG TTTTAACATT GATGCCAAAA CATGGAATTT GGTTAAAATT

1741 TCAAAAAATT GCTTAACTTT AGTTAAGAAT TATGTATTTG AACAAAAAAA AAATTAACAG

1801 TTCGTAACCT GAGAAAATTG TGTGGATTTT ACTATATATG ATAA
