# Supplementary material for: Transcription Factors AhR/ARNT Regulate the Expression of CYP6CY3 and CYP6CY4 Switch Conferring Nicotine Adaptation
Source: Int J Mol Sci. 2019 Sep 12;20(18):4521. doi: 10.3390/ijms20184521 (PMC6770377; doi:10.3390/ijms20184521)
Supplement: Supplementary file 1 [file ijms-20-04521-s001.zip › ijms-576626-supplementary data/Supporting Information Legends.docx]

Supporting Information Legends

Transcription factors AhR/ARNT regulate the expression of *CYP6CY3* and *CYP6CY4* switch conferring nicotine adaptation

Yiou Pan ^1,2^, Tianfei Peng ^2^, Pengjun Xu ^3^, Xiaochun Zeng ^2^, Fayi Tian ^2^, Jiabao Song ^2^, and
Qingli Shang ^1,2,^*

^1^ School of Agricultural Science, Zhengzhou University, Zhengzhou 450001 and PR China

^2^ College of Plant Science, Jilin University, Changchun 130062 and PR China

^3^ Institute of Tobacco Research, Chinese Academy of Agricultural Sciences, Qingdao 266101, China

***** Correspondence: shangqingli@163.com; Tel.: +86-431-87835745

**Supporting Information Legends**

**Table S1. Primers used in experiments**

**Fig. S1.** The *CYP6CY3* promoter, 5’-UTR and 5’-most coding sequences in *A*. *pisum*.

The nucleotides are numbered relative to the transcription start site (TSS) indicated by +1, with upstream sequences preceded by “-” and downstream sequences by “+”. The TATA box and other putative *cis* elements are underlined. The start codon ATG is shown in bold.

**Fig. S2.** The *CYP6CY3* promoter, 5’-UTR and 5’-most coding sequences in *A. gossypii*.

The nucleotides are numbered relative to the transcription start site (TSS), indicated by +1, with upstream sequences preceded by “-” and downstream sequences by “+”. The TATA box and other putative *cis* elements are underlined. The start codon ATG is shown in bold.

**Fig. S3. The promoter, 5’-UTR and 5’-most coding sequences of *CYP6CY4* of *M. persicae*.**

The nucleotides are numbered relative to the transcription start site (TSS) indicated by +1, with sequence upstream of it preceded by “-”, and downstream of it preceded by “+”. The TATA box and other putative *cis* elements are underlined. The start codon ATG is showed in bold.

**Fig. S4.** Functional characterization of *CYP6CY3* regulation by *AhR-ARNT* in *A. gossypii*.

(**A**) The relative transcript levels of *CYP6CY3* in the spirotetramat-resistant and susceptible cotton aphids [28]. (**B**) Orally mediated dsRNA knockdown efficacy (100 ng/μL of corresponding dsRNA) after 48 h and their effects on *CYP6CY3* transcriptional regulation in spirotetramat-resistant aphids. Different letters on the bars of the histogram indicate significant differences based on ANOVA followed by Tukey’s HSD multiple comparison test (P < 0.05). **Significant difference by Student’s t-test (P < 0.01).

**Supplementary Data 1. The expression of *CYP6CY3* and *CYP6CY4* in three races identified**

FPKM: Fragment per kilobases per million reads. The clean reads obtained in this study were submitted to the NCBI/SRA database (SRA experiment accession number: SRX1499035) (Peng et al., 2016) [23]

**Supplementary Data 2. CE/HPLC-MS identification of proteins isolated from the DNA pulldown.**

**Supplementary Data 3. Sequences of *AhR*, *ARNT*, *Hsp90*, *Camp*, *CncC* and *CYP6CY4* genes.**
