# Supplementary material for: Transcription Factors AhR/ARNT Regulate the Expression of CYP6CY3 and CYP6CY4 Switch Conferring Nicotine Adaptation
Source: Int J Mol Sci. 2019 Sep 12;20(18):4521. doi: 10.3390/ijms20184521 (PMC6770377; doi:10.3390/ijms20184521)
Supplement: Supplementary file 1 [file ijms-20-04521-s001.zip › ijms-576626-supplementary data/Table S1.docx]

**Table S1. Primers used in experiments**

| Primer name | Sequence (5'-3') | Application |
| --- | --- | --- |
| CYP6F  CYP6R  MpActF  MpActR  AphF  AphR  Cnc-F  Cnc-R  ARNT-F  ARNT-R  AhR-F  AhR-R  Camp-F  Camp-R  Hsp90-F  Hsp90-R  6CY4-F  6CY4-R  GenomeWalker adaptor  AP1  AP2  GSP1  GSP2  New GSP1  New GSP2  6CY4-GSP1  6CY4-GSP2  6CY3-2230-MluI 6CY3-998-MluI  6CY3-XhoI  6CY3-903-MluI  6CY3-573-MluI  ACdelF  ACdelR  Mut1F  Mut1R  Mut2F  Mut2R  AC_n_-F-MluI  AC_n_-R-XhoI  Ds6CY3-F1  Ds6CY3-R1  Ds6CY3-F2  Ds6CY3-R2  Ds6CY4-F1  Ds6CY4-R1  Ds6CY4-F2  Ds6CY4-R2  DsECFP-F1  DsECFP-R1  DsECFP-F2  DsECFP-R2  DsARNT-F1  DsARNT-R1  DsARNT-F2  DsARNT-R2  DsAhR-F1  DsAhR-R1  DsAhR-F2  DsAhR-R2  Dshsp90-F1  Dshsp90-R1  Dshsp90-F2  Dshsp90-R2  DsCnc-F1  DsCnc-R1  DsCnc-F2  DsCnc-R2  DsCamp-F1  DsCamp-R1  DsCamp-F2  DsCamp-R2  DsCnc-F1  DsCnc-R1  DsCnc-F2  DsCnc-R2  Cnc-F-AscI  Cnc-R-XhoI  ARNT-AscI  ARNT-PmeI  AhR-AscI  AhR-PmeI  Pulldown-F  Pulldown-R  Cnc-ORF-F  Cnc-ORF-R  ARNT-ORF-F  ARNT-ORF-R  Hsp90-ORF-F  Hsp90-ORF-R  AhR-ORF-F  AhR-ORFR  Camp-ORFpart-F  Camp-ORFpart-R  6CY3-ORF-F1  6CY3-ORF-R1  6CY3-ORF-F2  6CY3-ORF-R2 | CGGGGTGACGATCATCTATT  GGGTGGTCTTTTGACAAAGC  GGTGTCTCACACACAGTGCC  CGGCGGTGGTGGTGAAGCTG  TGGTATACACGTTGGTTCTC  GACCACGAGCTTCCCCGGTG  CGCCAGGATTTCGTGTG  TTCCACTGTTGCCCGTT  TTAGTTGCTATTGGTCGGTTG  CAAGTATATGTGTAACACGCTGA  GCAGCGTAGTTTCACCATCC  TGACTTCCTTGTGCGGTATTT  TGTACCTTCAGGGCTTTTC  CTTTGGTTCAATCCATTTCC  GGGAAGAACGAAAAGCAA  AATCCAGAATCCACACAGC  CAGATCAAAGAGTGCGGAGACG  CAAAAACGCAAGTGCCGATG  GTAATACGACTCACTATAGGGCACGCGTGGTCGACGGCCCGGGCTGGT  3’-NH2-CCCGACCA-PO4-5’  GTAATACGACTCACTATAGGGC  ACTATAGGGCACGCGTGGT  GTCCTCATCTGGAACAGTCCTCCGTAT  CAGTCGGTGGTTGACGATGATAGATTC  GTCCTCATCTGGAAYARTCCWCCGTAT  TCGGCCTGATGTAGGGCACGTTGAGTT  TGCCCAAGGCTATGTTCAAAAAGTTC  ATGCCGTAACGACATTTATCCACCAA  ACGCGTATTTATGACTATAACGAGG ACGCGTAGCTTTAGTATTGGATTGAA  CTCGAGCAGTCGGTGGTTGACGATGAT  ACGCGTATTATAATTTTTATGCGAGT  ACGCGTGTAACTTCAATACAATGATT  ATGTGTGGACACCAAATGCG  GTTACCGAATGTACCTACCT  CTCAGAGTCTCTCTGTGAGACTCTCGAGGTTATACGTCATAA  ATGTGTGGACACCAAATGC  TCTCAGAGTGTCACTCTGTGAGAGTGAGTCTACCTCCACC ATGTTACCGAATGTACCTAC  CATACTCATACGCATACCCATACTCGCGCGAACACGTCATAA  ATGTGTGGACACCAAATGC  GGTATGCGTATGAGTATGGGTATGCGTATGTGCGACTTCC ATGTTACCGAATGTACCTAC  ACGCGTATACTCTCGTCTGAACGT  CTCGAGCCTTGGTAGACAGCAGAGAG  GGTGtaatacgactcactatagggCAGAAATAATCAACAACGT  CGAGTGAAAGAAGTCAGT  CAGAAATAATCAACAACGT  GGTGtaatacgactcactatagggCGAGTGAAAGAAGTCAGT  GGTGtaatacgactcactatagggATTGTGTTTGATGATTACTCCTG  CTGAACAAAGCAACCAAAGTAG  ATTGTGTTTGATGATTACTCCTG  GGTGtaatacgactcactatagggCTGAACAAAGCAACCAAAGTAG  GGTGtaatacgactcactataggTTACGCCAAGCTTGCATGCCT  ACTCCAGCAGGACCATGTGATC  TTACGCCAAGCTTGCATGCCT  GGTGtaatacgactcactataggACTCCAGCAGGACCATGTGATC  GGTGtaatacgactcactataggGGGAGAATCATTGCGAAAT  CAAATGAACCCACGTCGAG  GGGAGAATCATTGCGAAAT  GGTGtaatacgactcactataggCAAATGAACCCACGTCGAG  GGTGtaatacgactcactataggCGTGGGAAAGAGTAACCCA  GCAATGTGATGGACGGAGA  CGTGGGAAAGAGTAACCCA  GGTGtaatacgactcactataggGCAATGTGATGGACGGAGA  GGTGtaatacgactcactataggTCGAGGAAGCACTAAGTG  CTGTTTCTCAGCTAATTGT  TCGAGGAAGCACTAAGTG  GGTGtaatacgactcactataggCTGTTTCTCAGCTAATTGT  GGTGtaatacgactcactataggGGCTCTTCACAGTTTAACCG  TGTTGCTTGGACCGAGG  GGCTCTTCACAGTTTAACCG  GGTGtaatacgactcactataggTGTTGCTTGGACCGAGG  GGTGtaatacgactcactatagggCCATTGTCACCTTGTTTT  GTTGATGTCGTTTTATTGC  CCATTGTCACCTTGTTTT  GGTGtaatacgactcactatagggGTTGATGTCGTTTTATTGC  GGTGtaatacgactcactataggGGCTCTTCACAGTTTAACCG  TGTTGCTTGGACCGAGG  GGCTCTTCACAGTTTAACCG  GGTGtaatacgactcactataggTGTTGCTTGGACCGAGG  GGCGCGCCATGTTGCGAATTAAAAAAGG  CTCGAGTTACTTGTCCTTGTGTTGCT  GGCGCGCCATGTACGGATACGCGGGTCCG  GTTTAAACTAGCTCATTCAAAATTTGTGTTG  GGCGCGCCGCGATGGCCGTGGCCGCGGT  GTTTAAACCCGCCCAGGTGACGGCGG  ATACTCTCGTCTGAACGT  CCTTGGTAGACAGCAGAGAG  ATGTTGCGAATTAAAAAAGGCT  TTACTTGTCCTTGTGTTGCTTGG  TAGTGTTTGTGTGTGTGTTACCGT  GTGTTGATAATACCTTACAGGCAGAT  GCATTGTGCTTGTACCCT  TCATGTCTTGTATGTAAACAGATA  CTGACATCGCAGGTTGCTC  GCGGGTGGTGACATTTTCTT  CCATTGTCACCTTGTTTT  GTTGATGTCGTTTTATTGC  AGTGTCGACTTGAGTGTGTAC  CCACACAATTTTCTCAGGTTACG  AGTGTGTACTGTGTATGGCGT  ACAATTTTCTCAGGTTACGAACTG | Real-Time  Real-Time  Real-Time  Real-Time  Real-Time  Real-Time  Real-Time  Real-Time  Real-Time  Real-Time  Real-Time  Real-Time  Real-Time  Real-Time  Real-Time  Real-Time  Real-Time  Real-Time  GenomeWalker  GenomeWalker  GenomeWalker  GenomeWalker  GenomeWalker  GenomeWalker  GenomeWalker  GenomeWalker  GenomeWalker  GenomeWalker  Make constructs  Make constructs  Make constructs  Make constructs  Make constructs  Make constructs  Make constructs  Make constructs  Make constructs  Make constructs  Make constructs  Make constructs  Make constructs  dsRNA synthesis  dsRNA synthesis  dsRNA synthesis  dsRNA synthesis  dsRNA synthesis  dsRNA synthesis  dsRNA synthesis  dsRNA synthesis  dsRNA synthesis  dsRNA synthesis  dsRNA synthesis  dsRNA synthesis  dsRNA synthesis  dsRNA synthesis  dsRNA synthesis  dsRNA synthesis  dsRNA synthesis  dsRNA synthesis  dsRNA synthesis  dsRNA synthesis  dsRNA synthesis  dsRNA synthesis  dsRNA synthesis  dsRNA synthesis  dsRNA synthesis  dsRNA synthesis  dsRNA synthesis  dsRNA synthesis  dsRNA synthesis  dsRNA synthesis  dsRNA synthesis  dsRNA synthesis  dsRNA synthesis  dsRNA synthesis  dsRNA synthesis  dsRNA synthesis  Make constructs  Make constructs  Make constructs  Make constructs  Make constructs  Make constructs  DNA pulldown  DNA pulldown  PCR  PCR  PCR  PCR  PCR  PCR  PCR  PCR  PCR  PCR  PCR  PCR  PCR  PCR |
