# Supplementary figures and images for: Transcription Factors AhR/ARNT Regulate the Expression of CYP6CY3 and CYP6CY4 Switch Conferring Nicotine Adaptation
Source: Int J Mol Sci. 2019 Sep 12;20(18):4521. doi: 10.3390/ijms20184521 (PMC6770377; doi:10.3390/ijms20184521)

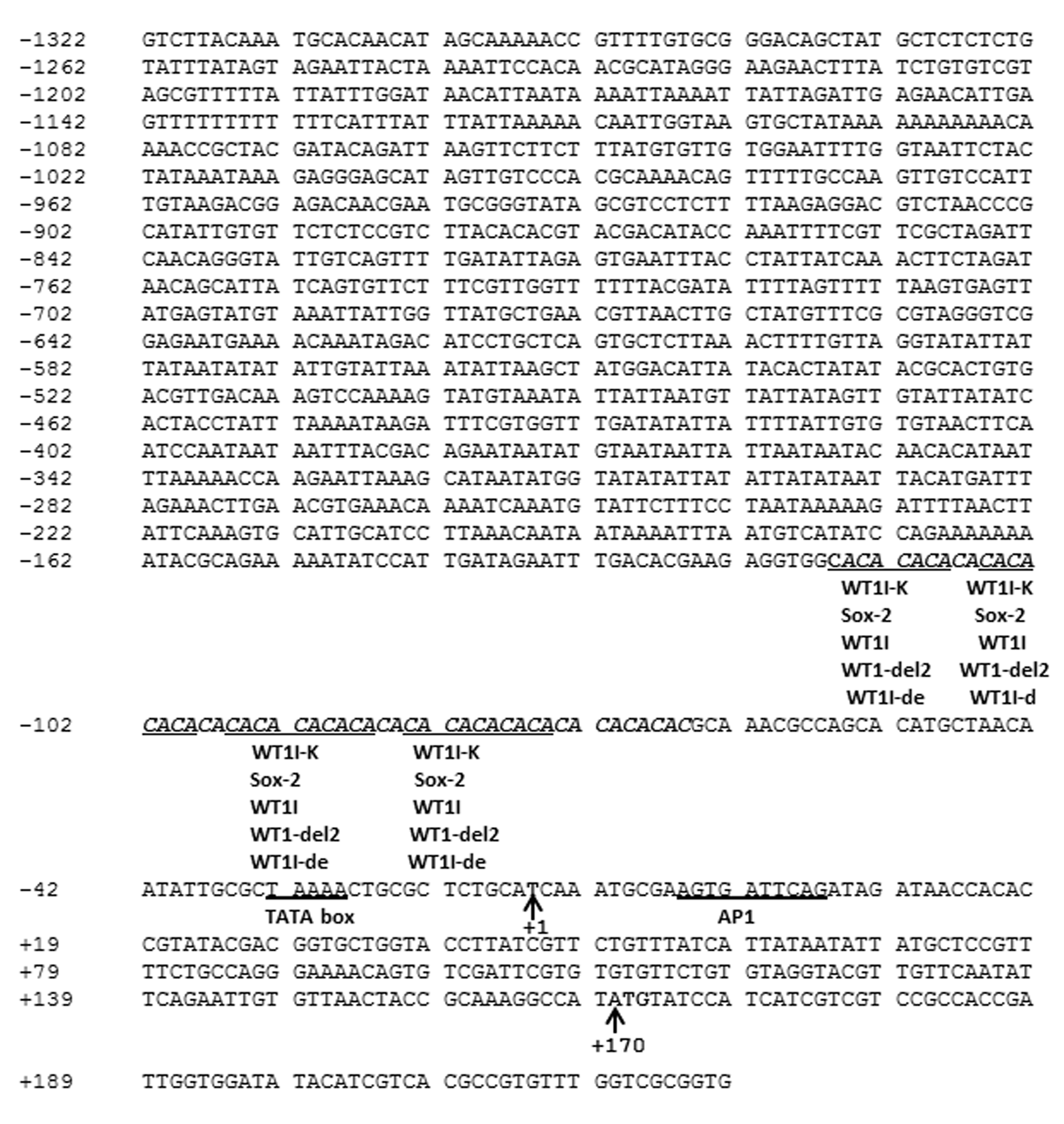

Supplement: Supplementary file 1 [file ijms-20-04521-s001.zip › ijms-576626-supplementary data/Figure S1.tif]

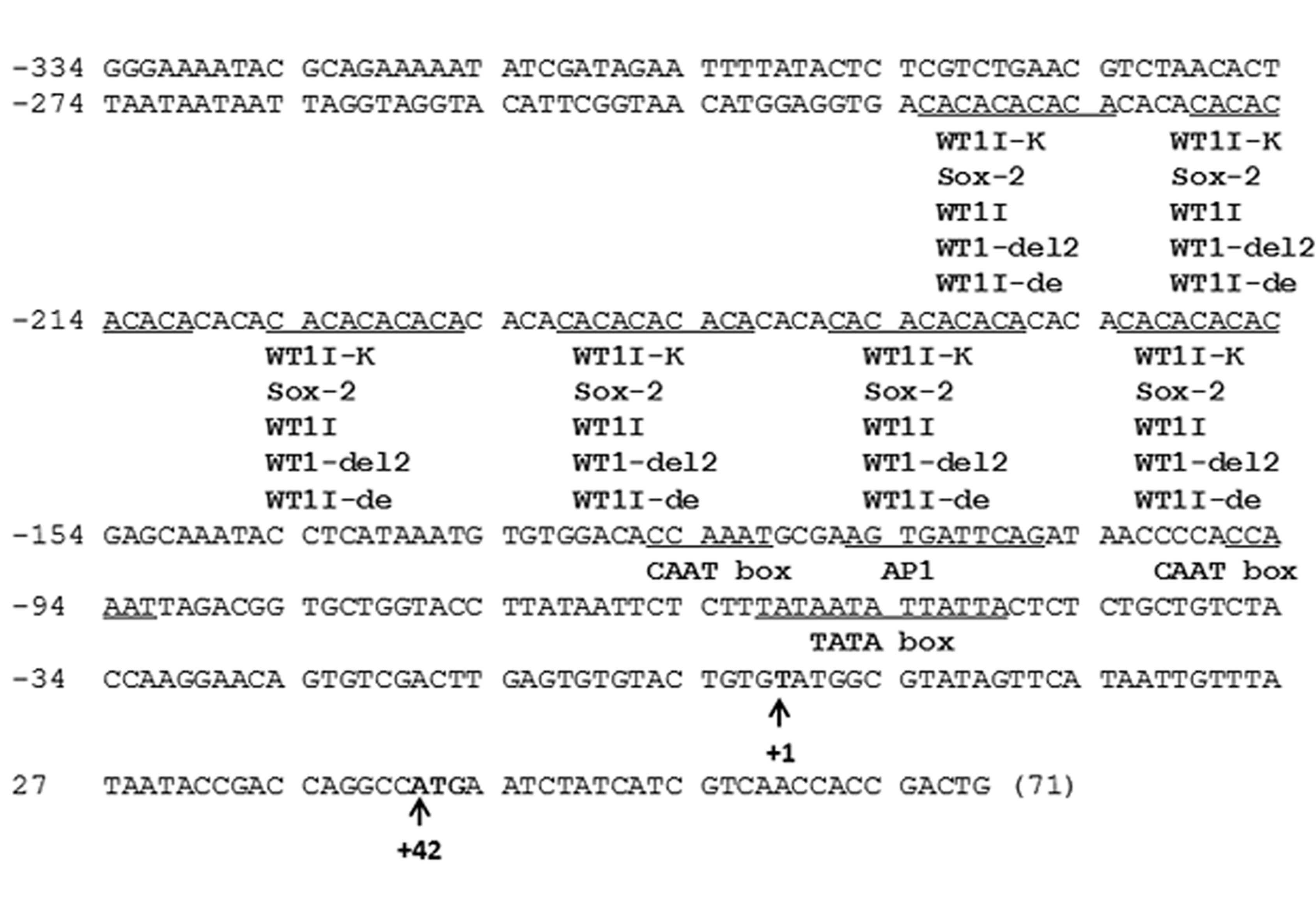

Supplement: Supplementary file 1 [file ijms-20-04521-s001.zip › ijms-576626-supplementary data/Figure S2.tif]

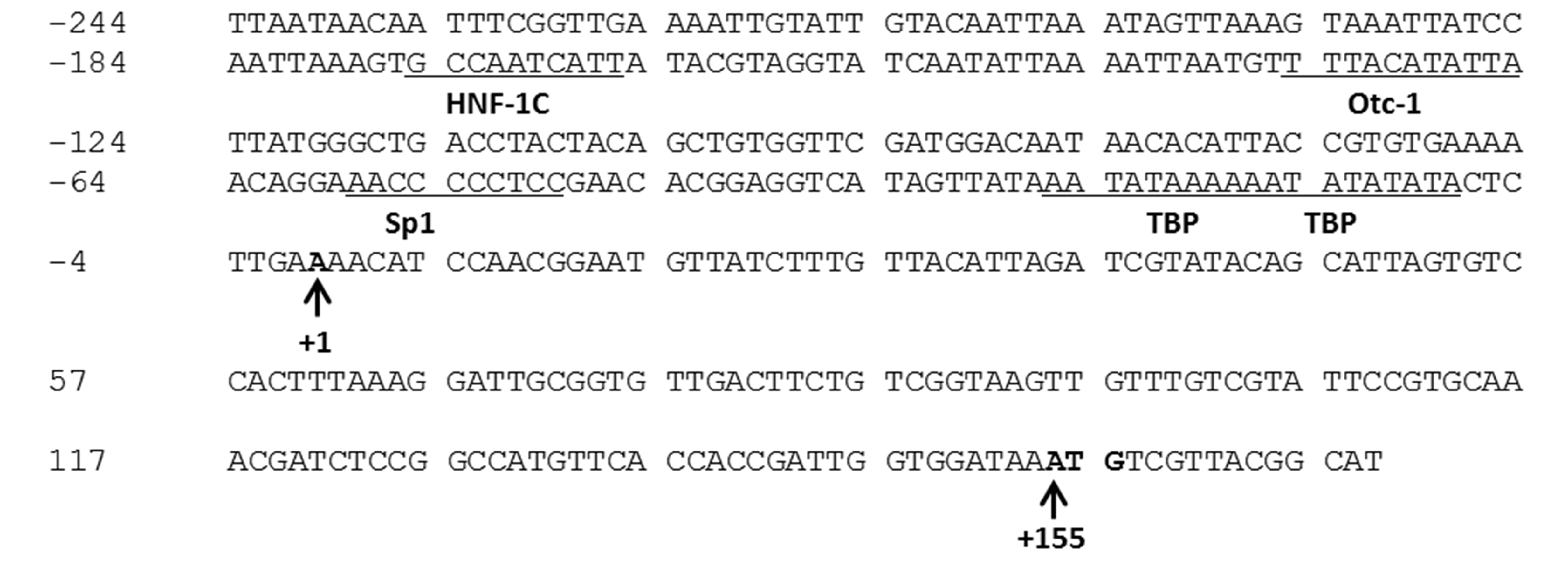

Supplement: Supplementary file 1 [file ijms-20-04521-s001.zip › ijms-576626-supplementary data/Figure S3.tif]

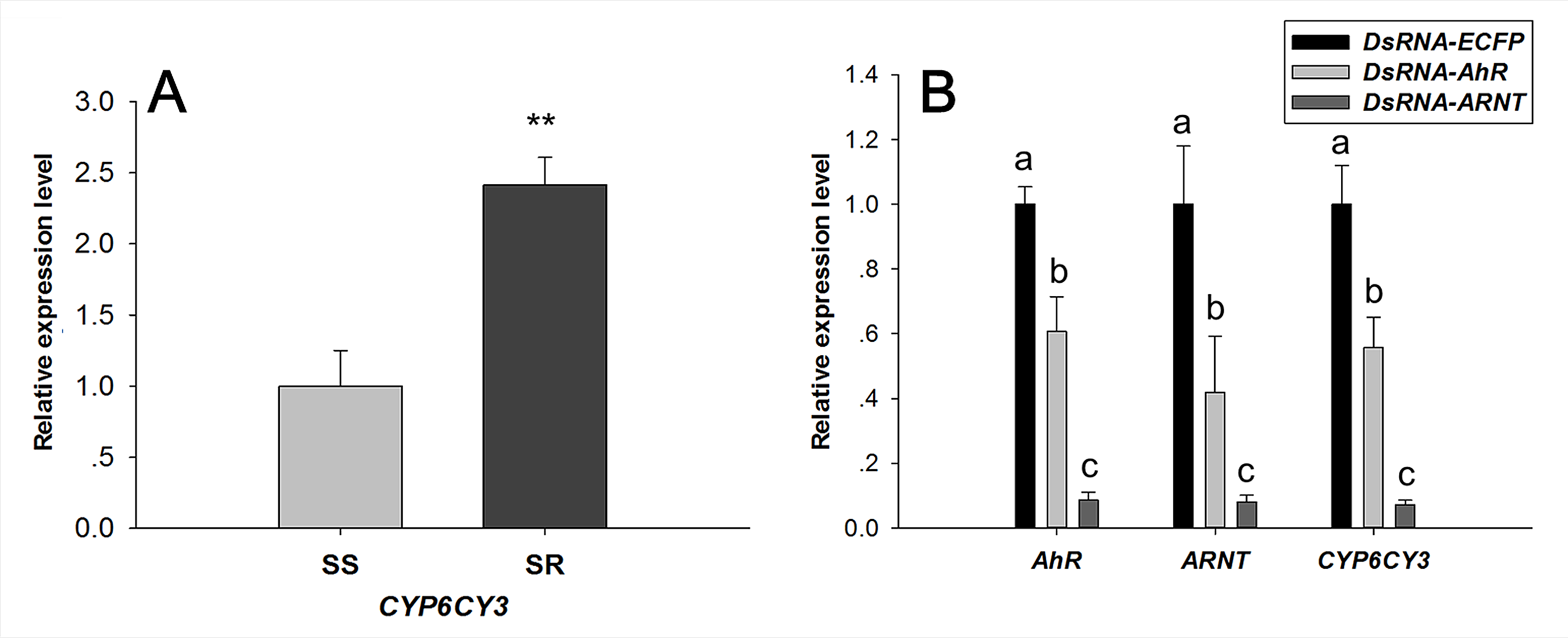

Supplement: Supplementary file 1 [file ijms-20-04521-s001.zip › ijms-576626-supplementary data/Figure S4.tif]
